# Identification of the sweet orange (*Citrus sinensis*) *bHLH* gene family and the role of *CsbHLH55* and *CsbHLH87* in regulating salt stress

**DOI:** 10.1002/tpg2.20502

**Published:** 2024-08-31

**Authors:** Yinqiang Zi, Mengjie Zhang, Xiuyao Yang, Ke Zhao, Tuo Yin, Ke Wen, Xulin Li, Xiaozhen Liu, Hanyao Zhang

**Affiliations:** ^1^ Key Laboratory for Forest Resources Conservation and Utilization in the Southwest Mountains of China Ministry of Education Southwest Forestry University Kunming China; ^2^ Key Laboratory of Biodiversity Conservation in Southwest China, National Forest and Grassland Administration Southwest Forestry University Kunming China

## Abstract

Salt stress is one of the primary environmental stresses limiting plant growth and production and adversely affecting the growth, development, yield, and fruit quality of *Citrus sinensis*. *bHLH* (basic helix‐loop‐helix) genes are involved in many bioregulatory processes in plants, including growth and development, phytohormone signaling, defense responses, and biosynthesis of specific metabolites. In this study, by bioinformatics methods, 120 *CsbHLH*genes were identified, and phylogenetic analysis classified them into 18 subfamilies that were unevenly distributed on nine chromosomes. The cis‐acting elements of the *CsbHLH* genes were mainly hormone‐related cis‐acting elements. Seventeen *CsbHLH* genes exhibited significant differences in expression under salt stress. Six *CsbHLH* genes with significant differences in expression were randomly selected for quantitative real‐time polymerase chain reaction (qRT‐PCR) validation. The qRT‐PCR results showed a strong correlation with the transcriptome data. Phytohormones such as jasmonic acid (JA) are essential for biotic and abiotic stress responses in plants, and *CsbHLH55* and *CsbHLH87* are considered candidate target genes for sweet orange *MYC2* transcription factors involved in the JA signaling pathway. These genes are the main downstream effectors in the JA signaling pathway and can be activated to participate in the JA signaling pathway. Activation of the JA signaling pathway inhibits the production of reactive oxygen species and improves the salt tolerance of sweet orange plants. The *CsbHLH55* and *CsbHLH87* genes could be candidate genes for breeding new transgenic salt‐resistant varieties of sweet orange.

## Core Ideas

- One hundred and twenty *CsbHLH* genes were identified from the sweet orange genome.
- Seventeen *CsbHLH* genes were significantly differentially expressed under salt stress.
- Phytohormones such as abscisic acid (ABA) and jasmonic acid (JA) play key roles in resistance to salt stress.
- *CsbHLH55* and *CsbHLH87* were identified as MYC2 transcription factors involved in the JA signaling pathway.
- The involvement of the sweet orange *bHLH* gene in the regulatory mechanism of salt stress was revealed.

**Abbreviations**
ABA: abscisic acid
cDNA: complementary DNA
DEGs: differentially expressed genes
FDR: false discovery rate
FPKM: fragments per kilobase per million
GA: gibberellin
GO: gene ontology
JA: jasmonic acid
KEGG: Kyoto Encyclopedia of Genes and Genomes
MeJA: methyl jasmonate
qRT‐PCR: quantitative real‐time polymerase chain reaction
TF: transcription factor

## 1 INTRODUCTION

Sweet orange (*Citrus sinensis*) is a genus of citrus in the family Rutaceae (Yin et al., 2022). It accounts for more than half of citrus production, has high economic value, and is rich in vitamin C, carotenoids, polyphenols, and other nutrients that are beneficial to human health and have high nutritional value (De et al., 2020; Huang et al., 2022; K. Zhu et al., 2022). However, in recent years, with the increasing severity of soil salinization, the growth, development, yield, and fruit quality of sweet oranges have been adversely affected (Duan et al., 2022). Therefore, research on salt tolerance genes and germplasm resources in sweet oranges is crucial and urgent.

The main stress modes of salt in plants include osmotic stress and ionic toxicity, which lead to stomatal closure and reduced photosynthetic and transpiration rates (Arif et al., 2020). Salt stress affects up to 831 million hm^2^ of land and is one of the primary environmental stresses affecting plant growth (L. Zhu et al., 2024). Most citrus varieties are sensitive to salt, and when sweet oranges are grown in saline environments, plant growth is slow, peel thickness is reduced, ripening is delayed, and yields are reduced (Mahmoud et al., 2021). To survive and optimize their living conditions, plants must use physiological and biochemical processes to adapt to environmental stresses. These processes occur through the activation or inhibition of gene‐specific expression (X. Sun et al., 2018). Transcription factors (TFs) are gene‐specific expression factors that interact with cis‐acting elements to activate or repress the specific expression of genes related to environmental stresses to maintain normal plant life activities (Niu & Fu, 2022).

*bHLH* (basic helix‐loop‐helix) family genes, one of the most common TF families in plants, are involved in a variety of bioregulatory processes in plants, including growth and development, phytohormone signaling, defense responses, and the biosynthesis of specific metabolites (Hou et al., 2023; Xu et al., 2022). In addition to their roles in plant growth, development, flowering, and metabolic biosynthesis, many *bHLHs* play roles in signaling and responses to abiotic stresses, such as salinity, drought, low temperature, and nutrient deficiencies (Guo et al., 2021). Genome‐wide identification and analysis have been completed for a variety of plants, including *Arabidopsis thaliana* (X. Li et al., 2006), *Helianthus annuus* (J. Li et al., 2021), *Dendrobium nobile* (He et al., 2023), *Oryza sativa* (X. Li et al., 2006), *Populus tomentosa* (K. Zhao et al., 2018), *Malus domestica* (Mao et al., 2017), *Vitis vinifera* (P. Wang et al., 2018), and *Cucumis sativus* (J. Li et al., 2020). To date, most bHLH proteins in *A. thaliana* have been functionally validated, and their primary roles include the regulation of growth and development, hormone signaling (abscisic acid [ABA], jasmonic acid [JA], etc.), and response to various stresses (cold, salt, etc.) (J. Li et al., 2021).

The *MYC* TF family is one of the subfamilies of the *bHLH* gene family (Y. Cao et al., 2022), and many JA‐responsive genes, especially the *MYC2* TF, are target genes of *bHLH* TFs (He et al., 2023). *bHLH* TFs play vital roles in the JA signaling pathway, which is crucial for orchestrating the adaptive response of plants to abiotic stresses (J. Wang et al., 2020). JASMONATE‐ZIM (JAZ) proteins and the *bHLH* subbranch are correlated with systemic JA responses (He et al., 2023). *MYC2* orchestrates the precise templates of the F‐box proteins CORONATINE INSENSITIVE1 (COI1) and JAZ to participate in JA signaling (Peñuelas et al., 2019). Moreover, overexpression of the bHLH gene can also enhance plant resistance to abiotic stresses. For example, in 2014, W. Liu et al. (2014) showed that overexpression of the *AtbHLH122* gene increased salt tolerance, osmoregulation, and proline concentration in *A. thaliana*. In 2020, Verma et al. (2020) reported that the expression of the *AtMYC2* gene in *A. thaliana*, which is activated by mitogen‐activated protein kinase (MAPK) and associated with elevated levels of proline, increased salt tolerance in *A. thaliana* plants, which is related to elevated proline levels. In the same year, J. Li et al. (2020) reported that the *CsbHLH041* gene in cucumber enhanced ABA‐induced salt tolerance in *A. thaliana* and cucumber seedlings, and Waseem et al. (2019) showed that overexpression of the *Lycopersicon esculentum SlbHLH22* gene enhanced the salt tolerance of tomato seedlings and enhanced the reactive oxygen species (ROS) clearing and osmoregulation abilities. More *bHLH* family genes have been identified in plants, and the understanding of *bHLH* TFs has gradually deepened, and the biological functions of their genes have been elucidated. However, few relevant studies on sweet orange *bHLH* TFs have been reported.

This study aimed to comprehensively investigate *bHLH* TF family members in the whole genome of sweet orange by identifying them, analyzing their physicochemical properties, performing phylogenetic analyses and gene structure analyses, and performing conserved motif analyses, analyses of cis‐acting elements, chromosomal localization, and gene duplication events, and gene ontology (GO) and Kyoto Encyclopedia of Genes and Genomes (KEGG) enrichment analyses. The expression pattern of these genes in sweet orange plants under salt stress was analyzed via transcriptional‐metabolic associations, and the mechanism of action of *bHLH* TFs involved in JA signaling and salt tolerance was explored. This study lays a foundation for understanding the evolution and biological functions of the *bHLH* TF family in sweet oranges and provides a basis for further breeding new salt‐tolerant sweet orange varieties.

**Core Ideas**

- One hundred and twenty *CsbHLH* genes were identified from the sweet orange genome.
- Seventeen *CsbHLH* genes were significantly differentially expressed under salt stress.
- Phytohormones such as abscisic acid (ABA) and jasmonic acid (JA) play key roles in resistance to salt stress.
- *CsbHLH55* and *CsbHLH87* were identified as MYC2 transcription factors involved in the JA signaling pathway.
- The involvement of the sweet orange *bHLH* gene in the regulatory mechanism of salt stress was revealed.

## 2 MATERIALS AND METHODS

### 2.1 Plant material

In this study, sweet orange seeds from sweet orange orchards in Xinping County, Yuxi City, Yunnan, China, were used as experimental materials, and the seeds were grown into complete plants via histoculture technology. Sweet orange is a citrus plant that accounts for more than 50% of the total citrus production. It is the most vital cultivar in the citrus industry, is rich in vitamin C and other nutrients, and has high economic and nutritional value (De et al., 2020; Huang et al., 2022; Yin et al., 2022). Thirty uniformly growing citrus plants were randomly selected, 15 of which were cultured in 2% NaCl media for 14 days. The remaining 15 plants were used as controls and were cultured in normal media.

### 2.2 Data acquisition

The genomic data for sweet orange (*C. sinensis* v3.0) were obtained from the Citrus CPBD (http://citrus.hzau.edu.cn/) genome database (Yin et al., 2022). Sequence information for the conserved structural domain of the *bHLH* TF family (ID: PF00010) was downloaded from the Pfam website (https://pfam.xfam.org/) (H. Sun et al., 2015). The protein sequences and genomic data of 158 *A. thaliana* *bHLH* TF families were downloaded from the *A. thaliana* genome website TAIR (https://www.Arabidopsis.org/) (L. Wang et al., 2019) (Table S1). The rice and *Citrus maxima* genomic data were downloaded from the rice genome database JGI (https://riceome.hzau.edu.cn/) and the Citrus genome database Citrus (http://citrus.hzau.edu.cn/).

### 2.3 Identification and physicochemical properties of the *bHLH* gene in sweet orange

First, HMMER software (http://hmmer.org/) was used to search for protein sequences containing conserved structural domains of the *bHLH* TF family (ID: PF00010) in the sweet orange genome, after which the protein sequences with an *E*‐value < 1 × 10^−5^ were screened out (Potter et al., 2018). Second, the BLASTP program of TBtools software was applied to BLAST the sweet orange genomic protein data using the protein sequence of the *A. thaliana bHLH* gene family as a reference. Sequences with higher homology (*E*‐value < 1 × 10^−5^) with the protein sequences of the *A. thaliana bHLH* gene family were screened from the sweet orange genomic protein data. Then, the HMMER software hmmsearch program was used to search for the conserved structural domains of the sweet orange protein sequences obtained by BLAST. Protein sequences with an *E*‐value < 1 × 10^−5^ were screened out. Finally, the protein sequences obtained in the above two steps were combined to get information on the sweet orange *bHLH* gene family. The presence of the *bHLH* structural domain was further confirmed using the CDD tool (https://www.ncbi.nlm.nih.gov/Structure/cdd/wrpsb.cgi). The sweet orange *bHLH* gene family members were obtained by removing the sequences of *bHLH* structural domains that were absent or incomplete. The genes were named according to their position on the chromosome with the nomenclature CsbHLH + sequence number. The physicochemical properties were analyzed using the online software ProtParam (http://www.expasy.org/tools/protparam.html).

### 2.4 Multiple sequence comparison and phylogenetic analysis

First, 120 *bHLH* sequences of sweet orange and 158 *bHLH* sequences of *A. thaliana* obtained from the identification were combined and used for pairwise sequence comparison and TBtools software trimming via MEGA11.0 software (Chen et al., 2020). Next, phylogenetic trees were constructed using the maximum likelihood method with MEGA11.0 software and IQtree. Finally, the evolutionary tree was built using the evolutionary tree beautification tool Chiplot (https://www.chiplot.online/), and the 120 sweet orange *bHLH* genes were grouped according to the grouping of *bHLH* genes in *A. thaliana*.

Amino acid sequence comparisons of bHLH members of the same subfamily were performed using MAFFT (version 7.5) comparison software, and the results and conserved regions were visualized using Geneious (version 9.1.4) and Jalview (version 2.11.2.7) comparisons (Katoh & Standley, 2013; Kearse et al., 2012; Waterhouse et al., 2009). The tertiary structures of the *A. thaliana* and sweet orange bHLH proteins were constructed using SWISS‐MODEL (https://swissmodel.ExPASy.org/).

### 2.5 Gene structure and conserved motif analysis

To further analyze the gene structure and the composition of conserved motifs in the sweet orange *bHLH* gene family, the gene structure of each gene was analyzed based on the sweet orange genome annotation file (GFF3) using TBtools software (Xi et al., 2023). The conserved motifs in the sweet orange *bHLH* genes were predicted using the online motif prediction tool MEME (https://meme‐suite.org/meme/). Visualization was subsequently performed using TBtools software.

### 2.6 Analysis of promoter cis‐acting elements

Nucleotide sequences 2 kb upstream of each *bHLH* gene were extracted from the sweet orange genome annotation file (GFF3) using TBtools software, and the online software PlantCARE (https://bioinformatics.psb.ugent.be/webtools/plantcare/html/) was used for promoter cis‐acting element analysis.

### 2.7 Chromosomal localization, duplicate genes, and covariance analysis

Chromosome position information was obtained from the genome annotation file (GFF3), and chromosome position mapping was performed with TBtools software. The chromosome length, position, and density information of the *CsbHLH* genes were extracted with TBtools, and the intraspecific covariance of the *CsbHLH* family members was analyzed via the MCSanX program in TBtools software. The GFF file gene sequence files of *A. thaliana*, rice, and apple were downloaded from the TAIR *A. thaliana* gene database, JGI rice gene database, and GDR Rosaceae gene database, respectively, and the interspecies covariance was constructed using TBtools software.

### 2.8 Transcriptome sequencing and determination of hormone content

Sweet orange leaves from the treatment and control groups were removed in liquid nitrogen for transcriptome and metabolome sequencing. The 15 salt‐stressed plants were randomly grouped and numbered T1, T2, and T3; the 15 normal‐growth plants were randomly numbered CK1, CK2, and CK3; and five samples were assigned to each numbered group. The leaves of each sample were removed as the experimental material sequentially. These samples were then stored in liquid nitrogen and transferred to Suzhou Panomics Biomedical Technology Co. for transcriptome sequencing and hormone content determination.

Transcriptome sequencing is mainly performed through RNA extraction, purification, and library construction, after which the libraries are subjected to paired‐end (PE) sequencing via next‐generation sequencing (NGS) via the Illumina sequencing platform. At present, the endogenous plant hormones that have been intensively studied are mainly categorized into nine major groups. In this study, phytohormone detection technology based on the liquid chromatography‐tandem mass spectrometry (LC‐MS/MS) platform was used to quantify 34 phytohormones, and the detected substances included the above nine categories of phytohormones.

Transcriptome data are available in the National Center for Biotechnology Information (nih.gov) (NCBI) database under accession numbers GSE248845, GSM7921250, GSM7921251, GSM7921252, GSM7921253, and GSM7921255.

The whole genome of sweet orange (SWO.v3.0.) was selected as the reference gene for the transcriptome sequencing data, and Bowtie2 software was used for sequence comparison. Then, using RSEM v1.3.0 software, the statistical results of Bowtie2 were compared to obtain the number of reads relative to each transcript for each sample, and the average expression level (fragments per kilobase per million [FPKM] bases) was calculated for each transcribed region. PE reads from the same fragment were counted as fragments, which provided the expression level EXPECTED (i.e., expected counts of transcripts) count data for genes and transcripts, where genes with FPKM values greater than 1.0 were considered to be expressed genes. The *p*‐value was determined by controlling the false discovery rate (FDR). Then, using the read count information, the differentially expressed genes (DEGs) were analyzed using the R language package edge R. The genes with the thresholds of FDR < 0.05, log_2_FC > 1, or log_2_FC < −1 were screened as the final DEGs.

### 2.9 Quantitative real‐time polymerase chain reaction (qRT‐PCR) analysis

To further verify the expression of the *CsbHLH14*, *CsbHLH16*, *CsbHLH17*, *CsbHLH49*, *CsbHLH50*, *CsbHLH55*, and *CsbHLH87* genes under salt stress. Sweet orange leaves cultured in regular media for 14 days were used as the control group (CK1, CK2, CK3), and kiwifruit leaves cultured in salt‐stressed medium for 14 days were used as the treatment group (T1, T2, T3). Total RNA was extracted from sweet orange leaves using a Plant RNA Extraction Kit (DP432) and reverse transcribed into complementary DNA (cDNA) using a FastKing RT Kit (KR116). cDNA was mixed with a 10‐fold dilution of reverse‐transcribed cDNA and mixed well for use in formal quantitative PCR experiments. The qRT‐PCR primers (Table S2) were designed using Primer 6.0 with β‐actin as the internal reference gene. Standard RT‐qPCR was then performed using a SYBR Green qPCR Master Mix kit with at least three replicates for each gene. The data were analyzed using SPASS v21.

## 3 RESULTS

### 3.1 Identification and physicochemical properties of the sweet orange *bHLH* gene family

**FIGURE 1:**
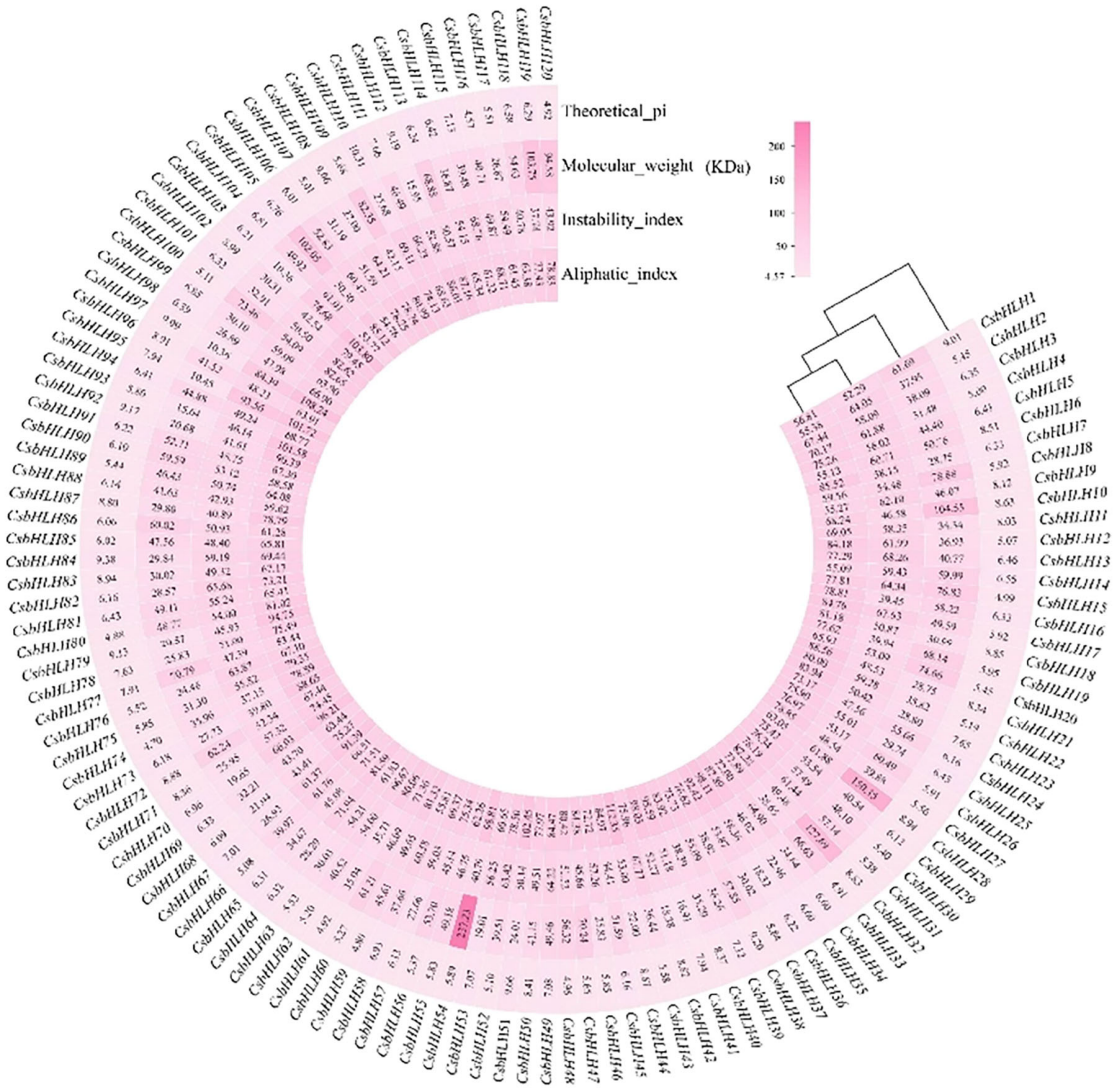
Physicochemical property analysis plot of the *CsbHLH* gene. The numbers in the heatmap indicate the numerical values; the higher the value is, the darker the color. The gene name is stated at the outermost circle, followed by the theoretical pi, molecular weight (kDa), instability index, and aliphatic index.

BLAST and hmmsearch searches were performed on the protein sequences of the sweet orange genome using the protein sequences of the *A. thaliana bHLH* gene family as a control. Next, the sweet orange *bHLH* gene family was predicted by CDD to remove incomplete sequences, and a total of 120 *bHLH* genes were ultimately identified. The genes were named *CsbHLH1‐bHLH120* according to their chromosomal position. The sweet orange bHLH protein sequences were further analyzed, and the structures are shown in Figure 1 and Table S3. The protein length of each *bHLH* gene varied widely, ranging from 91 (*CsbHLH97*) to 2142 (*CsbHLH54*) amino acids; the molecular weight ranged from 10.36 (*CsbHLH97*) to 237.23 (*CsbHLH54*) kDa; the isoelectric point ranged from 4.57 (*CsbHLH116*) to 10.31 (*CsbHLH110*); the total number of atoms ranged from 1467 (*CsbHLH103*) to 33531 (*CsbHLH54*); the instability index ranged from 35.71 (*CsbHLH61*) to 93.71 (*CsbHLH95*); and the lipolysis index ranged from 53.44 (*CsbHLH44*) to 112.35 (*CsbHLH77*).

### 3.2 Phylogeny of the sweet orange *bHLH* gene family

**FIGURE 2:**
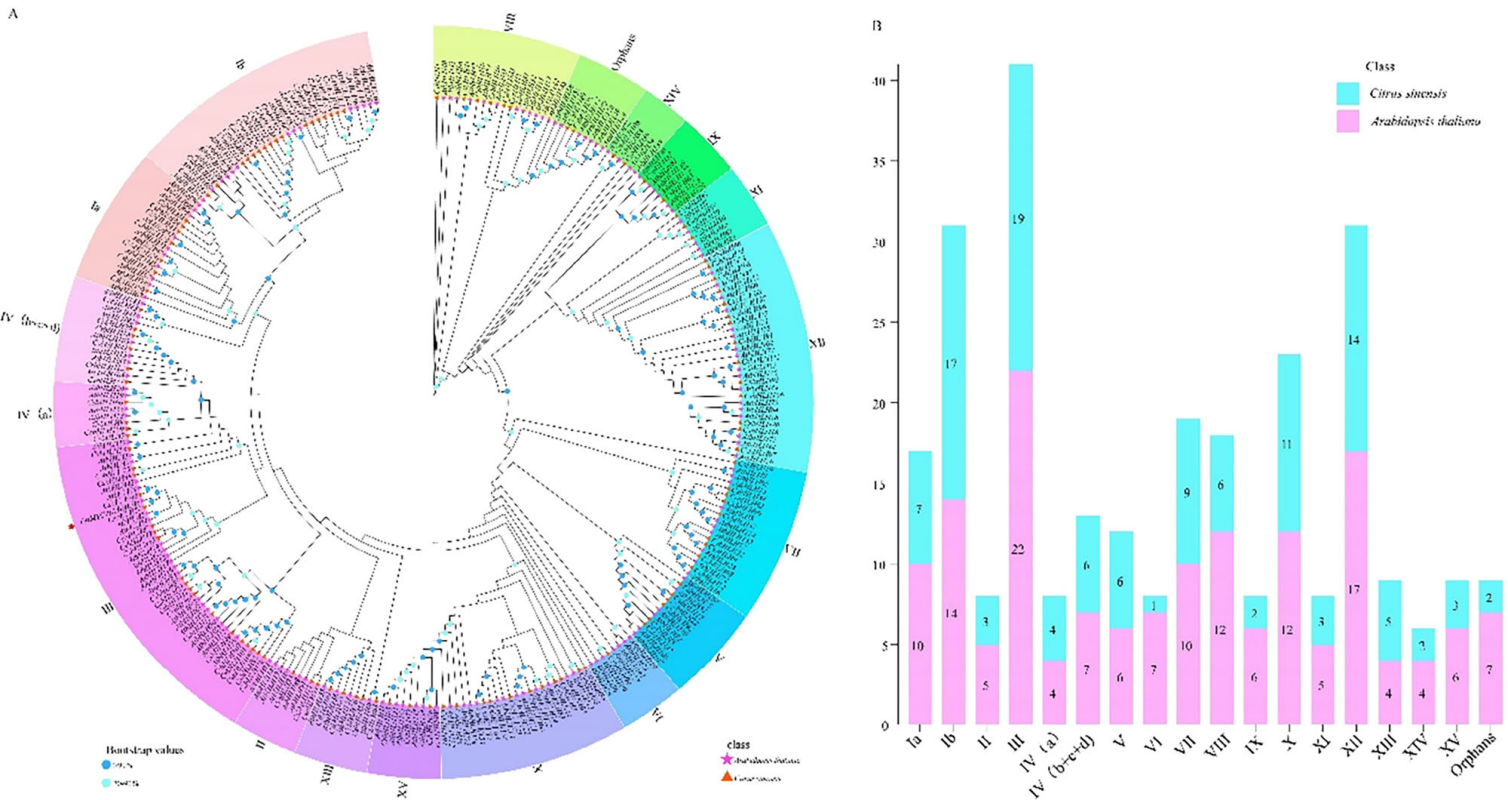
Phylogenetic tree of the *Arabidopsis thaliana* and sweet orange *bHLH* gene families. (A) Phylogenetic tree plot constructed by the maximum likelihood (ML) method using a total of 1000 replicated bootstrap values, with different subgroups indicated by different colors; *CsbHLH* genes indicated by orange pentagrams; blue triangles indicate *AtbHLH* genes in the plot; gray circles indicate self‐expansion values between 70% and 90%; black circles indicate self‐expansion values >90%; red pentagrams indicate *AtbHLH6* (*AtMYC2*); and *CsbHLH55* and *CsbHLH87* are clustered in the same subfamily as functionally validated AtCsbHLH6. (B) Plot of the number of *A. thaliana* and sweet orange *bHLH* genes in each subfamily, with blue denoting sweet orange and pink‒purple denoting *A. thaliana*.

Phylogenetic trees were constructed for the amino acid sequences encoded by 120 bHLH genes of sweet orange and 158 *bHLH* genes of *A. thaliana* to understand the relationships between the *bHLH* genes of different species. The clustering results showed (Figure 2A) that sweet orange *bHLH* genes were categorized into 18 subfamilies based on the 18 *bHLH* subfamilies of *A. thaliana* (J. Li et al., 2023), and each subfamily included *bHLH* genes from both species. According to the gene data of individual subfamilies (Figure 2B), subfamily III had the greatest number of genes (41), including 22 in *A. thaliana* and 19 in sweet orange; subfamily VI contained only one sweet orange *bHLH* gene (*CsbHLH94*); and subfamily XIV had the lowest number of genes and contained only six *bHLH* genes, including two *CsbHLH* genes and four *AtbHLH* genes. Except for a few genes, the clustering relationships of the evolutionary tree showed good agreement with the classification of *bHLH* genes in sweet orange, and genes with close relatives during the identification process were usually classified into the same subclade. In *A. thaliana*, the *AtbHLH6* gene is an *AtMYC2* TF gene, and overexpression of the *AtMYC2* gene enhances salt tolerance in *A. thaliana* (D. Verma et al., 2020). Moreover, in the phylogenetic tree, the *CsbHLH55* and *CsbHLH87* genes belong to subclade III with the *AtbHLH6* (*AtMYC2*) gene and are closely related, suggesting that the *CsbHLH55* and *CsbHLH87* genes may have similar functions.

### 3.3 Gene structure and conserved motif analysis of the *CsbHLH* gene

**FIGURE 3:**
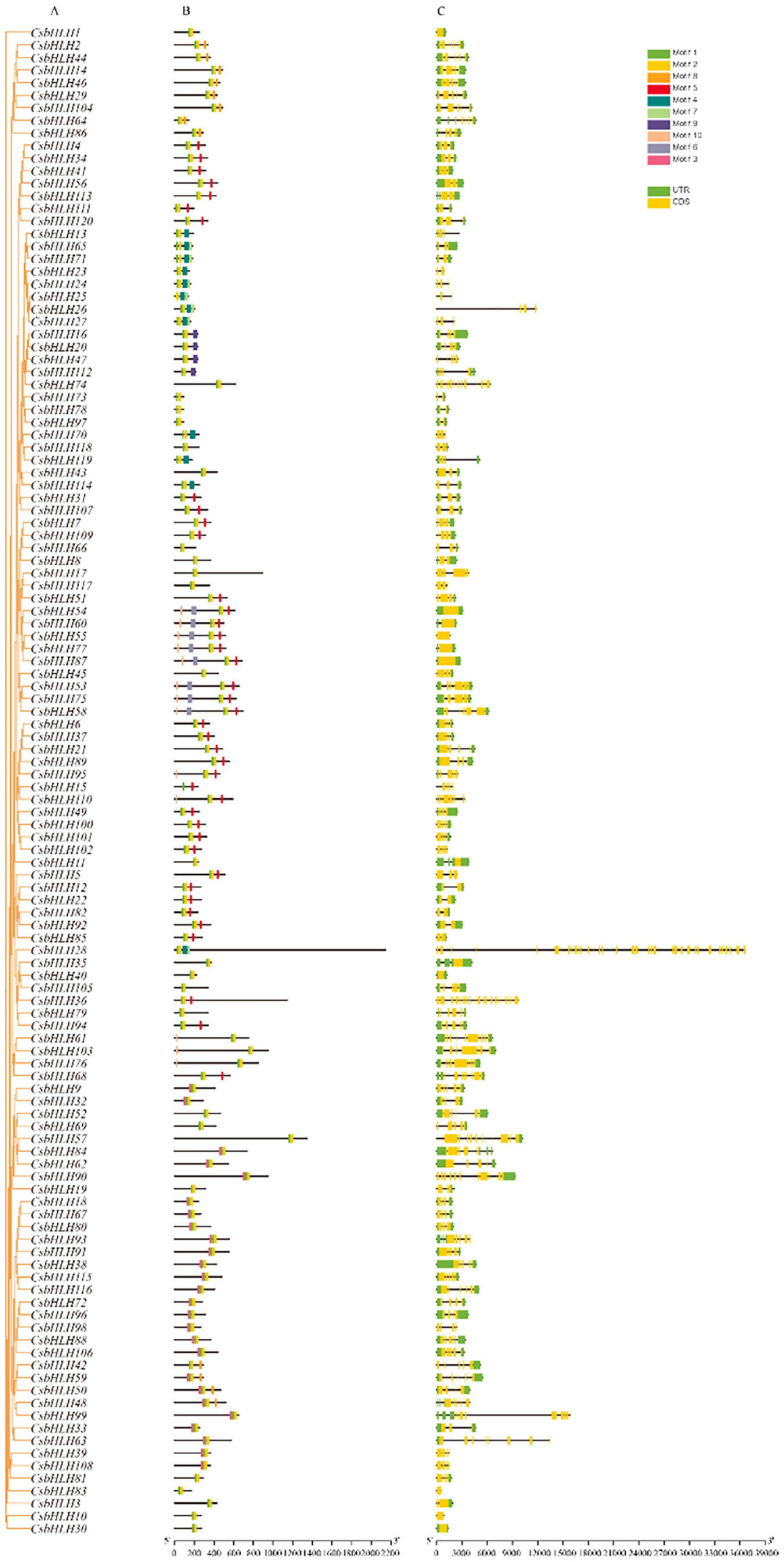
Conserved motifs and gene structure diagram of the *CsbHLH* gene family. (A) Phylogenetic tree of the sweet orange *bHLH* genes. (B) Distribution of conserved motifs in *CsbHLHs*. The motifs are represented by colored boxes, and the black lines indicate the relative lengths of the proteins. (C) Exon‐intron structure diagram of the *CsbHLH* gene. Green indicates the UTR, yellow indicates the CDS, and the line segments in the middle indicate the introns. CDS, coding sequence; UTR, untranslated region.

The motif composition of the protein sequence was analyzed in this study using the MEME online tool to investigate the amino acid sequence characteristics of the *CsbHLH* gene. As shown in Figure 3B, conserved motifs 1, 2, and 5 were highly conserved, but the same motif had different positions in different protein sequences, which might be related to the structure and function of the protein. Motif 9 appeared only in *bHLH16*, *bHLH20*, *bHLH47*, and *bHLH112*, suggesting that these genes might be involved in specific functions. Conversely, motifs 1 and 2 were present in all the *CsbHLH* genes, suggesting that these two motifs are highly conserved. According to the phylogenetic tree of *CsbHLHs* (Figure 3A), there were some differences in motif distribution among members of the same subfamily. For example, the phylogenetic analyses revealed that *CsbHLH42*, *CsbHLH50*, and *CsbHLH59* clustered together as belonging to the same subfamily, but motif 3 did not appear only in *CsbHLH42*, which may be due to differences in the TF families under specific conditions.

The distribution of conserved motifs in the *CsbHLH* gene family may also be influenced by gene structure. The determination of the distribution of gene structures is the key to studying evolution within gene families. Gene structure analysis of 120 *CsbHLH* genes revealed (Figure 3A,C) that for the 120 sweet orange *bHLH* genes, the majority (119) were less than 18 kb in length, and the remaining gene (*CsbHLH28*) was approximately 39 kb in length. The number of introns and exons in the sweet orange *bHLH* genes varied widely, with the number of introns ranging from 0 to 40 and the number of exons ranging from 1 to 41. Seven *CsbHLH* genes (*CsbHLH1*, *CsbHLH10*, *CsbHLH30*, *CsbHLH54*, *CsbHLH55*, *CsbHLH83*, and *CsbHLH87*) had only one exon and no introns. The *CsbHLH28* gene had the longest and greatest number of introns and exons.

### 3.4 Analysis of cis‐acting elements in the sweet orange *bHLH* gene family promoter

**FIGURE 4:**
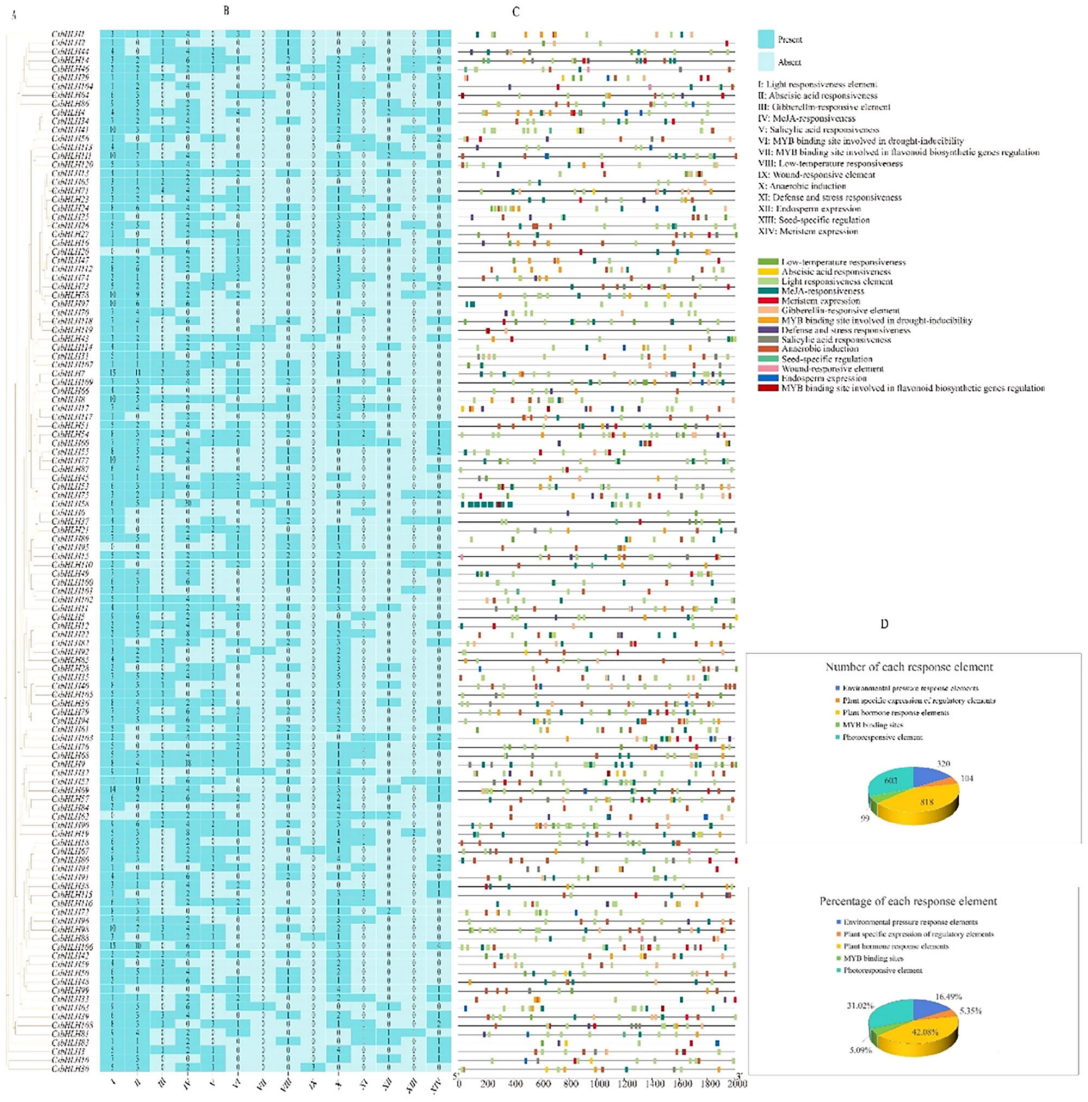
Cis‐acting elements in the sweet orange *bHLH* gene. (A) Phylogenetic tree of the sweet orange *bHLH* genes. (B) The number of cis‐acting elements for each bHLH gene; the numbers in the heatmap box indicate the number of different elements in these *CsbHLH genes*; (C) position of each cis‐acting element; and (D) category, number, and percentage of cis‐acting elements.

To understand the response mechanism of the sweet orange *bHLH* gene, 120 promoter cis‐acting elements 2000 bp upstream of the sweet orange *bHLH* gene were predicted using the online software PlantCARE. A total of 1944 promoter cis‐acting elements were identified (Figure 4A–C) and were divided into five major groups: phytohormone‐responsive elements, environmental stress‐responsive elements, light‐responsive elements, plant‐specific regulatory elements, and MYB‐binding sites. The percentages of these components were, in descending order, phytohormone‐responsive components (42.08%), environmental stress‐responsive components (31.02%), light‐responsive components (16.49%), plant‐specific regulatory components (5.35%), and MYB‐binding sites (5.09%) (Figure 4D). Combined with phylogenetic analysis of the *CsbHLH* genes (Figure 4A,B), genes clustered in the same subfamily contained cis‐acting elements that were also morphologically similar. In summary, 83.51% of promoter‐acting elements, in addition to light‐responsive elements, could regulate gene expression and metabolism in plants to increase plant resistance and improve maladaptation to adverse environments. Among them, phytohormone response elements are the most common, but the expression of *bHLH* genes is most likely to occur through phytohormone regulation when plants are subjected to external disturbances (J. Li et al., 2021).

### 3.5 Chromosomal localization and duplicate gene analysis of the sweet orange *bHLH* gene

**FIGURE 5:**
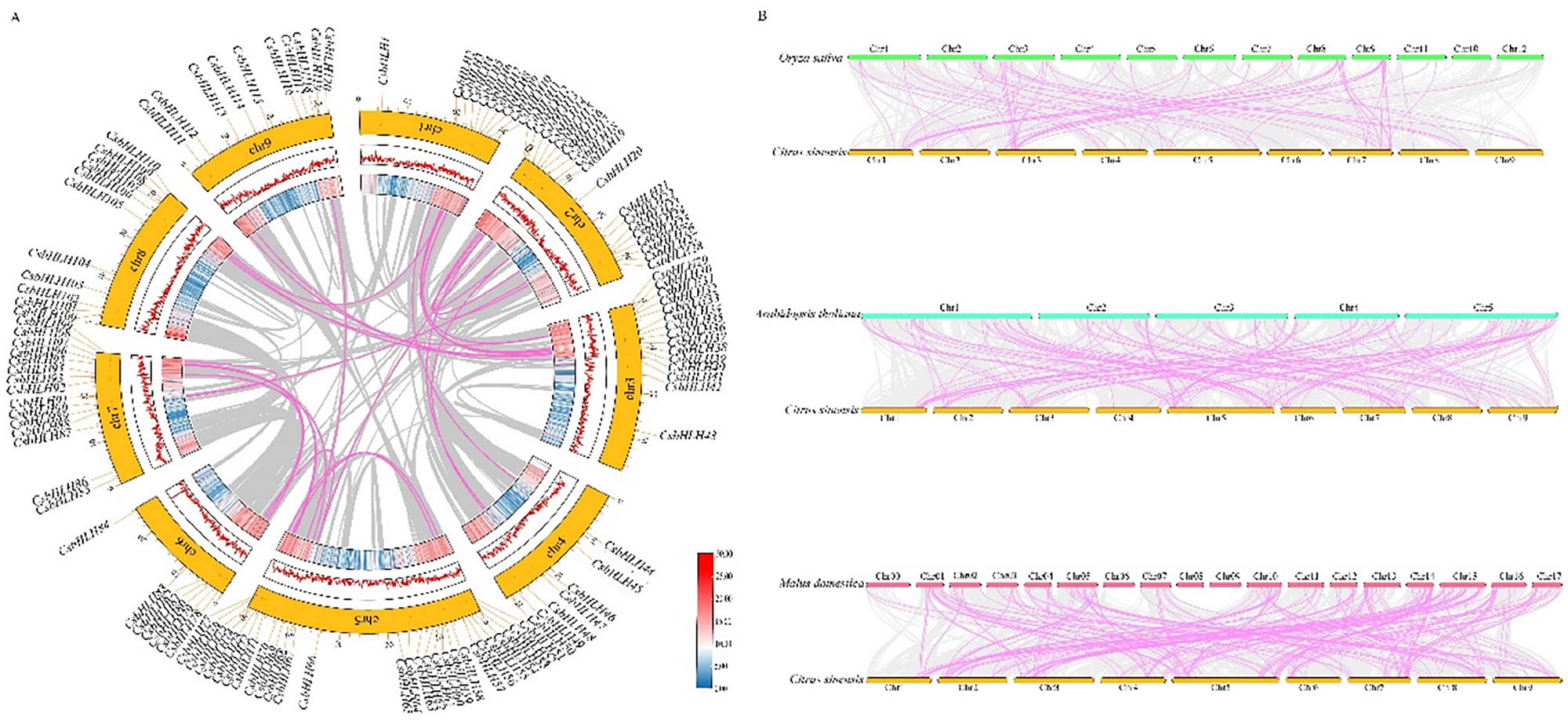
Sweet orange *bHLH* gene localization and covariance map. (A) Chromosomal localization map of the sweet orange *bHLH* gene. The gray line indicates all duplicated genes in the sweet orange genome, and the pink line indicates the genes duplicated in the sweet orange *bHLH* segment; the orange and yellow graphs indicate the chromosomes of sweet orange. (B) Collinearity map of different species of sweet orange, rice, *Arabidopsis thaliana*, and *Citrus maxima*. The gray lines indicate duplicated blocks, while the pink lines indicate collinear pairs of *bHLH* genes. The threshold for covariance analysis in this process is 10.

The results of chromosome localization (Figure 5A) showed that the 120 *bHLH* genes of sweet orange were unevenly distributed on nine chromosomes, with most of the genes being located on chromosome 5 (27) and the fewest genes being located on chromosome 6 (7). Gene duplication is a vital means of gene family expansion in plants. In this study, 28 segmental duplication gene pairs were identified among the 120 *CsbHLH* genes, with seven segmental duplication gene pairs on both chromosome 2 and chromosome 5 accounting for the greatest number of gene pairs and duplication pairs on the remaining chromosomes. These results suggest that segmental duplications are crucial for *CsbHLH* family expansion.

In addition, the covariance of *bHLH* genes among sweet orange, rice, *A. thaliana*, and *C. maxima* plants was analyzed in this study, and the results (Figure 5B) showed that sweet orange and *C. maxima* had the most homologous gene pairs, while sweet orange and rice had the fewest homologous gene pairs. These findings indicate that sweet oranges and *C. maxima* have a closer evolutionary relationship and that sweet oranges and rice have a more distant evolutionary relationship.

### 3.6 Analysis of phenotypic and physiological indices of sweet orange plants under salt stress

**FIGURE 6:**
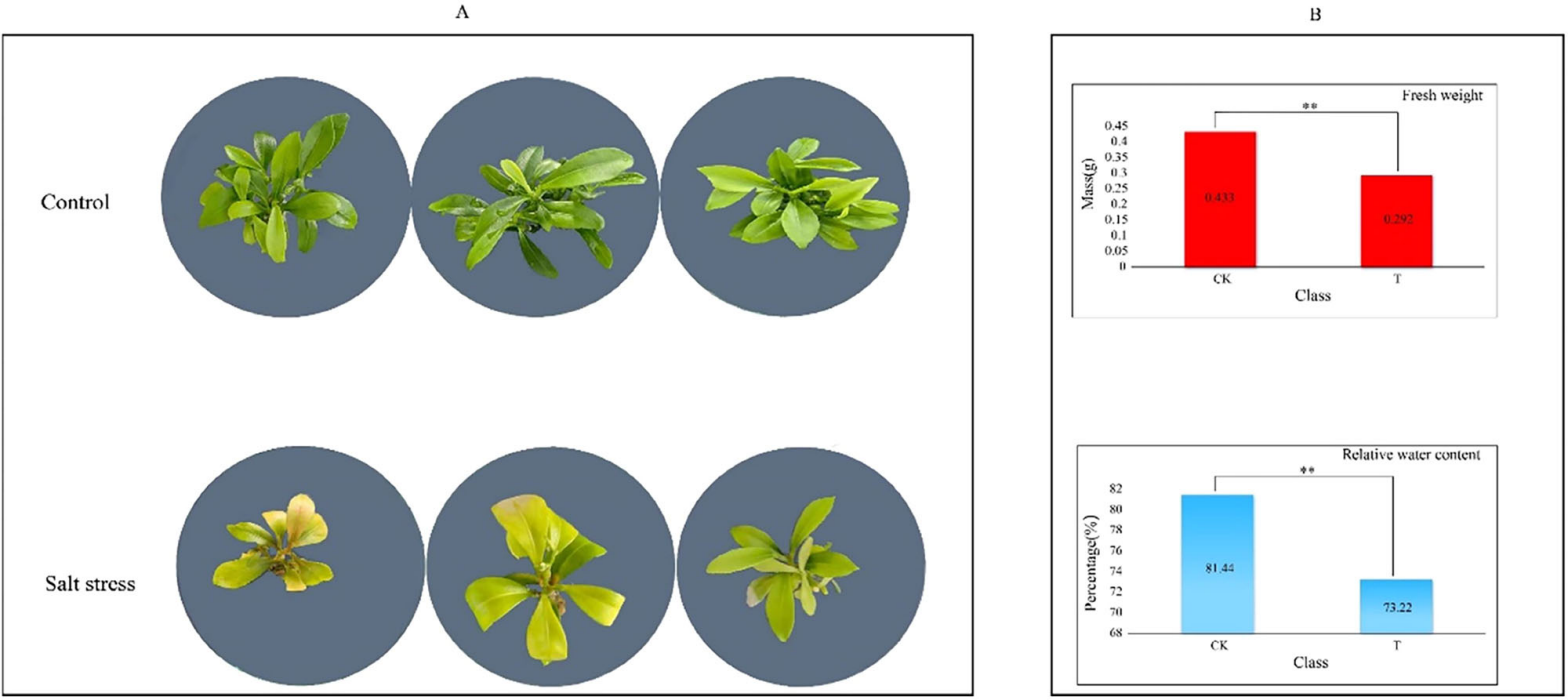
Phenotype and growth diagram of sweet orange plants under salt stress. (A) Phenotypic observations of sweet orange histocultured plants under control and salt stress conditions (14 days). Control indicates the control group, and salt stress indicates the treatment group. (B) Comparison of physiological indices of sweet orange histocultured plants under control and salt stress conditions (14 days). Red indicates fresh weight, blue indicates relative water content, CK: control group, T: treatment group; ^**^ *p* value < 0.01.

Salt stress is a primary environmental stress that affects plant growth and development, and salt stress enhances the osmotic pattern of the plant system and causes water loss in plants (S. Zhao et al., 2021). In this study, 30 sweet orange group‐cultured plants with approximately the same growth were divided into 15 plants each and cultured in media supplemented with 2% NaCl or the control for 14 days. The results showed (Figure 6; Table S6) that salt stress severely inhibited the growth of sweet orange seedlings compared to those in the control group and resulted in the yellowing, curling, and abscission of the sweet orange leaves (Figure 6A). Taken together, these findings suggest that plant growth, development, and physiological metabolism are severely disrupted under salt stress and that plants adapt to environmental changes by altering their morphology (Fang et al., 2021). After comparing the control and salt stress conditions for 14 days, the fresh weight and water content of the sweet orange plants under salt stress were significantly lower than those under the control conditions (*p *< 0.01) (Figure 6B). Taken together, these findings indicate that water flows out of plant cells under high salt stress, causing osmotic stress and physiological drought in plants (J. Sun et al., 2019).

### 3.7 Expression pattern and qRT‐PCR analysis of sweet orange *bHLH* genes under salt stress

**FIGURE 7:**
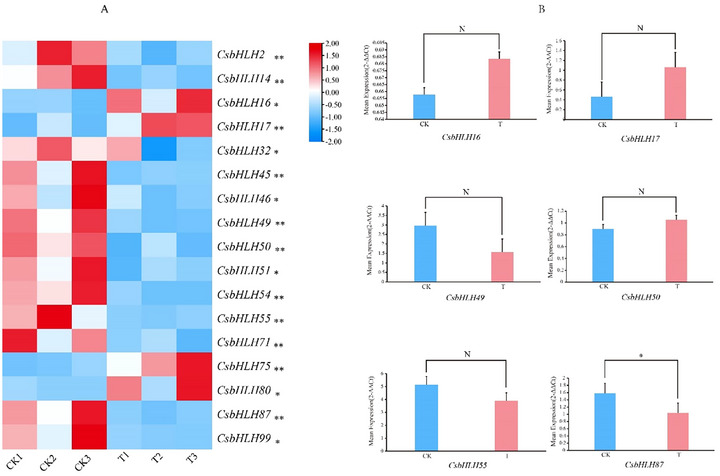
The expression patterns of CsbHLH under salt stress and quantitative real‐time polymerase chain reaction (qRT‐PCR) results were plotted. (A) Heatmap of the expression of CsbHLH DEGs under salt stress^*^ *p* value < 0.05; ^**^ *p* value < 0.01. (B) Plot of the qRT‐PCR results for the eight differentially expressed CsbHLH genes. The horizontal axis denotes the treatment group, CK denotes the control group, and T denotes the treatment group; the vertical axis denotes the average expression; ^N^ *p* value > 0.05; ^*^ *p* value < 0.05.

Transcriptome sequencing was performed on sweet orange leaves under salt stress for 14 days to determine the expression pattern of *CsbHLH* genes under salt stress for 14 days, and sweet orange seedlings under salt stress for 0 day were used as controls. A total of 17 out of the 120 *CsbHLH* genes were significantly differentially expressed under salt stress (14 days) compared with the control (CK); of these genes, only four (*CsbHLH16*, *CsbHLH17*, *CsbHLH75*, and *CsbHLH80*) were upregulated, while the remaining genes were downregulated. There were more downregulated DEGs than upregulated genes, suggesting that the *CsbHLH* genes may negatively regulate the damage caused by salt stress to sweet oranges and thus maintain their life activities (Figure 7A; Table S7).

qRT‐PCR was used for validation. As shown in Figure 7B and Table S8, the expression of *CsbHLH16*, *CsbHLH17*, and *CsbHLH50* was upregulated, and the expression of *CsbHLH54*, *CsbHLH55*, and *CsbHLH87* was downregulated under salt stress (14 days) compared with that of the control (CK). SPSS software was used to analyze the correlation between the FPKM values and qRT‐PCR results for the transcriptome data (Figure S1). The correlation coefficients were all greater than 0.6, which further confirmed the accuracy of the transcriptome data obtained via qRT‐PCR, and the correlation between the results of the six *CsbHLH* genes randomly selected as having significant differences in most of the transcriptome data and the transcriptome data was high. The accuracy of the transcriptome data was proven, and the reliability of the DEGs in the transcriptome data was verified. Moreover, these genes are vital for salt stress resistance in sweet oranges.

According to the transcriptome data, in addition to the *CsbHLH* genes, many other genes (Figure S2), such as the *Cs_ont_5g00425*0, *Cs_ont_1g019000*, *Cs_ont_7g027580*, *Cs_ont_1g018990*, *Cs_ont_4g022880*, and *Cs_ont_4g022240* genes, are also significantly differentially expressed under salt stress. These six genes may be involved in JA signaling, and their expression was significantly downregulated, indicating that these six genes may be coexpressed with *CsbHLH* genes.

### 3.8 GO functional enrichment analysis

**FIGURE 8:**
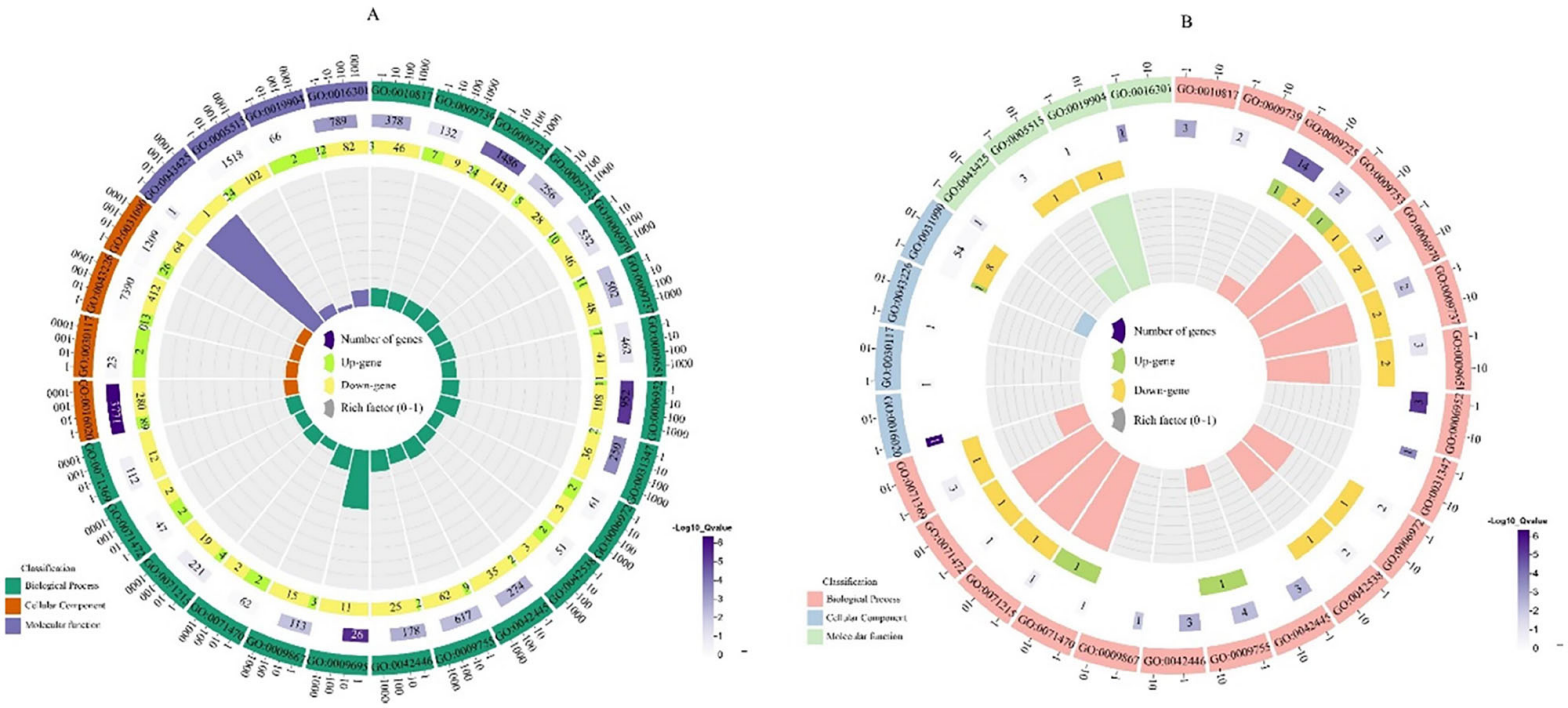
Gene ontology (GO) functional enrichment analysis plot. (A) GO functional enrichment of the whole genome under salt stress in sweet orange. The first circle bar represents the number of differentially expressed genes (DEGs) enriched in the same term as a proportion of the total number of genes; the second circle represents the number of DEGs enriched in GO terms upregulated and downregulated, with green indicating upregulated genes and yellow indicating downregulated genes; and the third circle heatmap represents the total number of genes enriched in the corresponding GO term. The fourth circle represents the GO number, and different categories are indicated by various colors. (B) GO functional enrichment of *bHLH* genes under salt stress in sweet orange. The first circle bar graph indicates the ratio of the number of *bHLH* DEGs enriched in the same GO term to the total number of *bHLH* genes; the second circle represents the number of DEGs upregulated and downregulated according to the GO term; the green color indicates upregulation; the yellow color indicates downregulation; and the third circle heatmap represents the total number of genes enriched in the corresponding GO term. The fourth circle represents the GO number, and different classifications are indicated by different colors.

GO enrichment analysis helps in understanding the potential molecular functions of genes encoding proteins and in further understanding the functions of genes. The DEGs were subjected to GO enrichment analysis based on the transcriptome data to understand the regulatory role of sweet orange genes under salt stress. As shown in Figure 8A, sweet orange was enriched in more genes related to hormone processes under salt stress, such as GO:0009753 (response to JA), GO:0009737 (response to ABA), GO:0009739 (response to gibberellin [GA]), and GO:0010817 (regulation of hormone levels). The biological processes associated with hormones are closely related to salt stress in sweet oranges.

To further elucidate the biological functions of the *CsbHLH* family, in this study, the proteins of 120 *CsbHLH* genes were functionally annotated using the EggNOG‐MAPPERS database, and GO functional enrichment analysis was performed in combination with transcriptome data. As shown in Figure 8B and Table S5, 120 *CsbHLH* genes were annotated into three categories: biological process, cellular component, and molecular function. Among the biological processes, most were related to hormone biological processes and osmoregulation; among the cellular components, most were related to biofilms. These findings suggested that the *CsbHLH* gene may adapt plants to adverse environments by regulating the levels of hormones, including JA, GA, and ABA, as well as by adjusting the osmotic potential in the cell body.

### 3.9 KEGG enrichment of the *CsbHLH* gene family and metabolite analysis of sweet orange plants under salt stress

**FIGURE 9:**
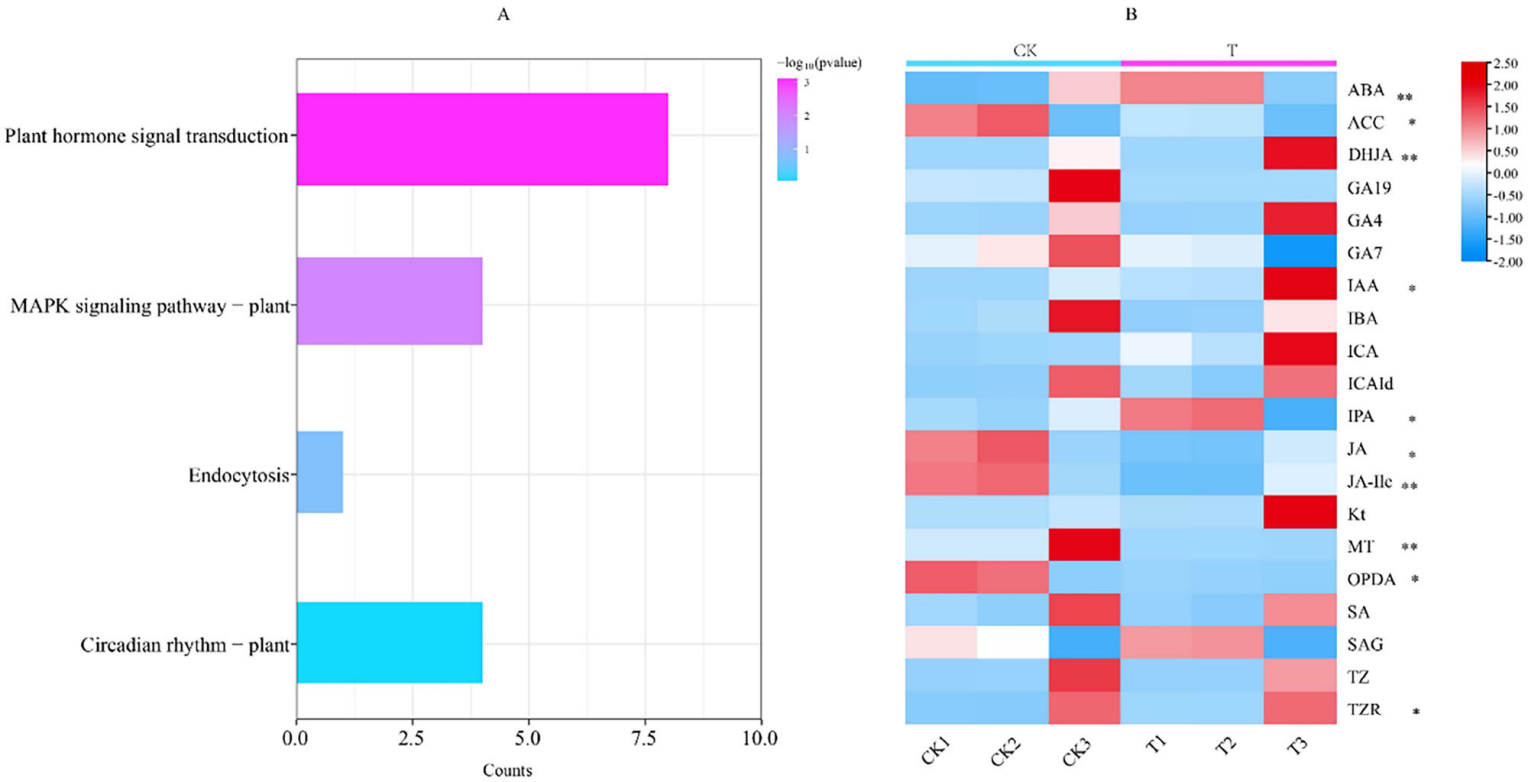
Heatmap of the Kyoto Encyclopedia of Genes and Genomes (KEGG) pathways associated with *CsbHLH* and changes in hormone content in salt‐stressed sweet orange plants. (A) KEGG enrichment plot of the *CsbHLH* genes. The horizontal axis indicates the number of enriched genes, the vertical axis indicates the KEGG pathway type, and the different colors indicate different *p* values. (B) Heatmap of hormone contents in sweet orange plants under salt stress. CK, control group; T, treatment group; ^*^ *p* value < 0.05; ^**^ *p* value < 0.01. MAPK, mitogen‐activated protein kinase.

KEGG enrichment analysis was subsequently performed to determine the biological functions of the *CsbHLH* genes under salt stress. As shown in Figure 9A and Table S9, eight *CsbHLH* genes were enriched in plant hormone signal transduction, and four *CsbHLH* genes were enriched in the MAPK signaling pathway (MAPK signaling pathway—plant) under salt stress. Combined with the transcriptome data (Figure 9A), the DEGs *CsbHLH55* and *CsbHLH87* were enriched for plant hormone signaling and MAPK signaling pathway functions. It has been shown that cells can respond to many stresses, including salt stress, by regulating the MAPK cascade and that hormones such as JA influence signaling through the MAPK cascade (Jagodzik et al., 2018).

Phytohormones are critical for helping plants adapt to unfavorable environmental conditions such as salt and drought (V. Verma et al., 2016). The content of hormone metabolites under salt stress in sweet oranges was determined based on targeted secondary metabolomics to understand the role of hormones in salt stress regulation in sweet oranges. As shown in Figure 9B and Table S10, the levels of 20 hormones were altered in sweet orange plants under salt stress (14 days) compared with those in the control (CK) plants. The contents of ABA, dihydro jasmonic acid (DHJA), growth hormone (IAA), indole propionic acid (IPA), and zeatin riboside (TZR) were significantly increased. However, the contents of ethylene (ACC), JA, jasmonic acid‐isoleucine (JA‐Ile), melatonin (MT), and 20‐dodecenoic acid (OPDA) decreased significantly. These findings indicate that phytohormones in sweet oranges play crucial roles in the response to salt stress.

**FIGURE 10:**
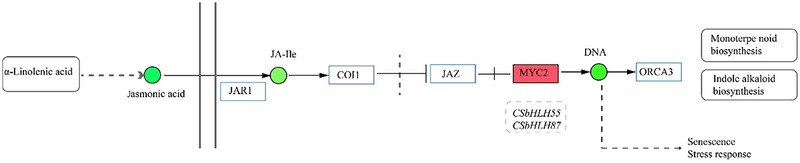
Diagram of the Kyoto Encyclopedia of Genes and Genomes (KEGG) phytohormone transduction pathway in response to salt stress for the *CsbHLH55* and *CsbHLH87* genes. The red box indicates the process of regulation of the MYC2 protein in response to jasmonic acid (JA).

KEGG enrichment analysis (Figure 10) revealed that the CsbHLH55 and CsbHLH87 genes encode MYC2 TFs in the JA signaling pathway. Under salt stress, α‐linolenic acid forms JA through several transformations, and JA forms JA‐Ile via jasmonic acid‐amino acid synthase (JAR1). In the absence of hormone signaling, the JAZ protein represses the MYC2 TF, which binds to the cis‐acting element of the JA response gene. JA stimulates the specific binding of the JAZ protein to COI1, which induces the degradation of JAZ. The degradation of JAZ removes the repression of the MYC2 TF and allows the expression of the JA response gene, enhancing the plant resistance.

As shown in Figure 9B, CK3 and T3 were not reproducible compared with the other samples, which may be related to the live seedlings used in this study, and the differences in the genetic background of the live seedlings may have led to the differences in the experimental results. Therefore, the metabolite contents were analyzed for significant differences, and the results revealed strong differences in the variation in hormone contents.

### 3.10 Multiple sequence comparisons and three‐dimensional protein structures of proteins encoded by the *CsbHLH55*, *CsbHLH87*, and *AtMYC2* (*AtbHLH6*) genes

**FIGURE 11:**
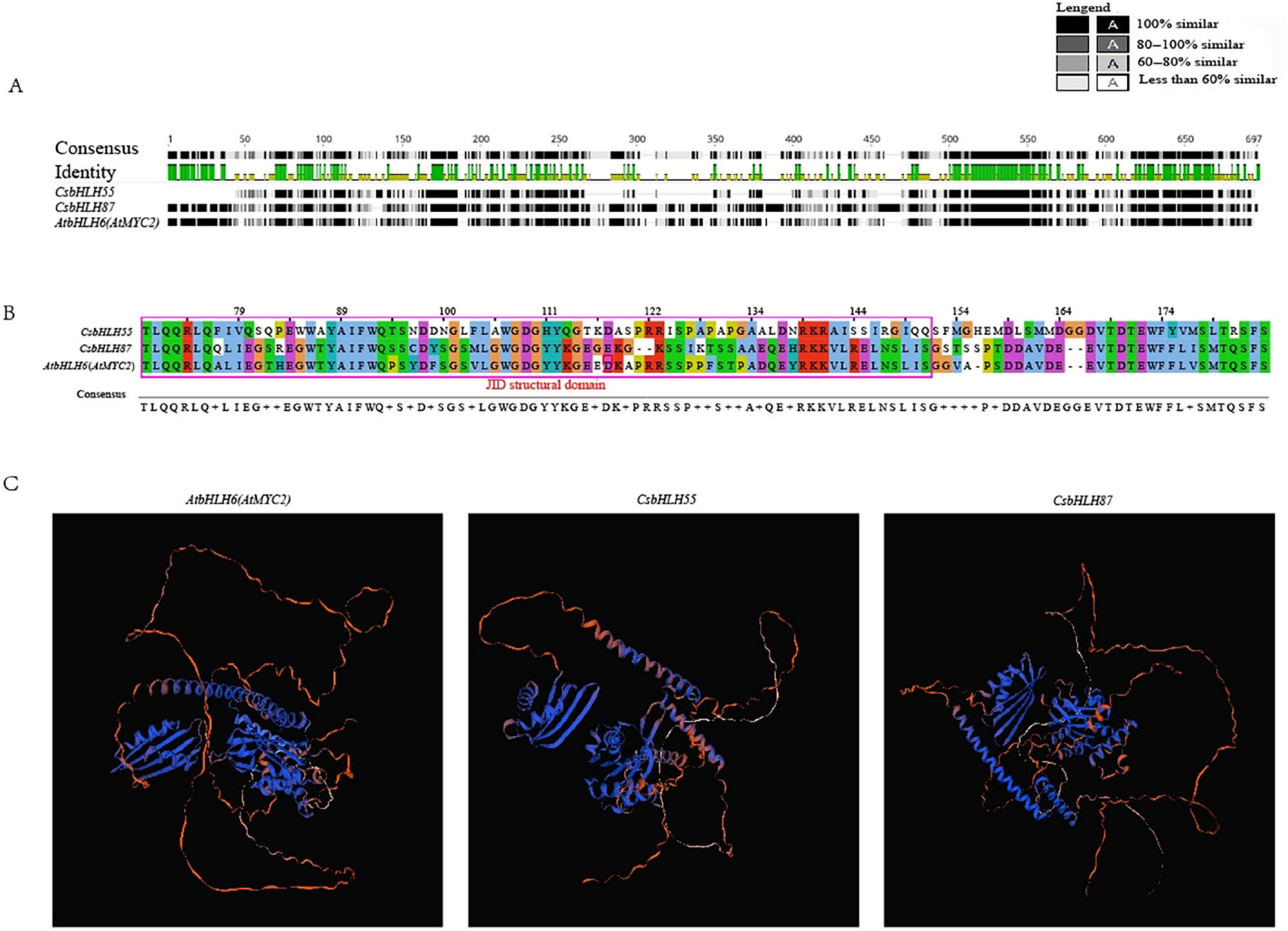
Sequence comparison and tertiary structure maps of the *CsbHLH55*, *CsbHLH87*, and *AtMYC2* proteins. (A) Multiple comparisons of protein sequences encoded by the *CsbHLH55*, *CsbHLH87*, and *AtMYC2* genes, with black and gray shading indicating identical and similar amino acid residues, respectively. (B) Multiple comparisons of the active site regions and specific substrate selection switch regions of *CsbHLH55*, *CsbHLH87*, and *AtMYC2*; the rectangular boxes indicate the JID structural domains particular to the *MYC2* gene. (C) Three‐dimensional structures of the *CsbHLH55*, *CsbHLH87*, and *AtMYC2* proteins.

The protein sequences encoded by the *CsbHLH55*, *CsbHLH87*, and *AtMYC2* (*AtbHLH6*) genes were comparatively analyzed. The results showed (Figure 11A) that these three genes are highly conserved with high protein sequence similarity, suggesting that their functions may be similar. The MYC proteins belong to the bHLH family and are distinguished from other bHLHs by the presence of the JID structural domain. The presence of JID domains in all three genes (*CsbHLH55*, *CsbHLH87*, and *AtMYC2* [*AtbHLH6*]) identified in this study (Figure 11B) suggested that the *CsbHLH55* and *CsbHLH87* genes are *MYC* genes. Proteins with similar structural sequences have conserved three‐dimensional structures, and the conserved structural domains of different proteins have conserved functions. The protein tertiary structures of *CsbHLH55*, *CsbHLH87*, and *AtMYC2* were constructed using SWISS‐MODEL software. The results showed (Figure 11C) that the protein structures of the two genes were highly similar, and the ratios of α‐helices, β‐folds, elongated strands, and free coiling were very close. This indicates that the structural domains of these two proteins are very similar and have conserved functions.

### 3.11 JA‐responsive genes are target genes of *bHLH* transcription factors (*MYC2*)

JA regulates plant growth and development and is crucial for biotic and abiotic stress responses (Wasternack & Hause, 2013). JA‐Ile is the biologically active form of JA that induces the formation of receptor complexes, leading to ubiquitination and degradation of the JAZ transcriptional repressor (Thines et al., 2007). Under environmental stress, JAZ can interact with the F‐box COI1, which forms a coreceptor complex, leading to JAZ degradation and transcriptional induction (F. Zhang et al., 2015).

**FIGURE 12:**
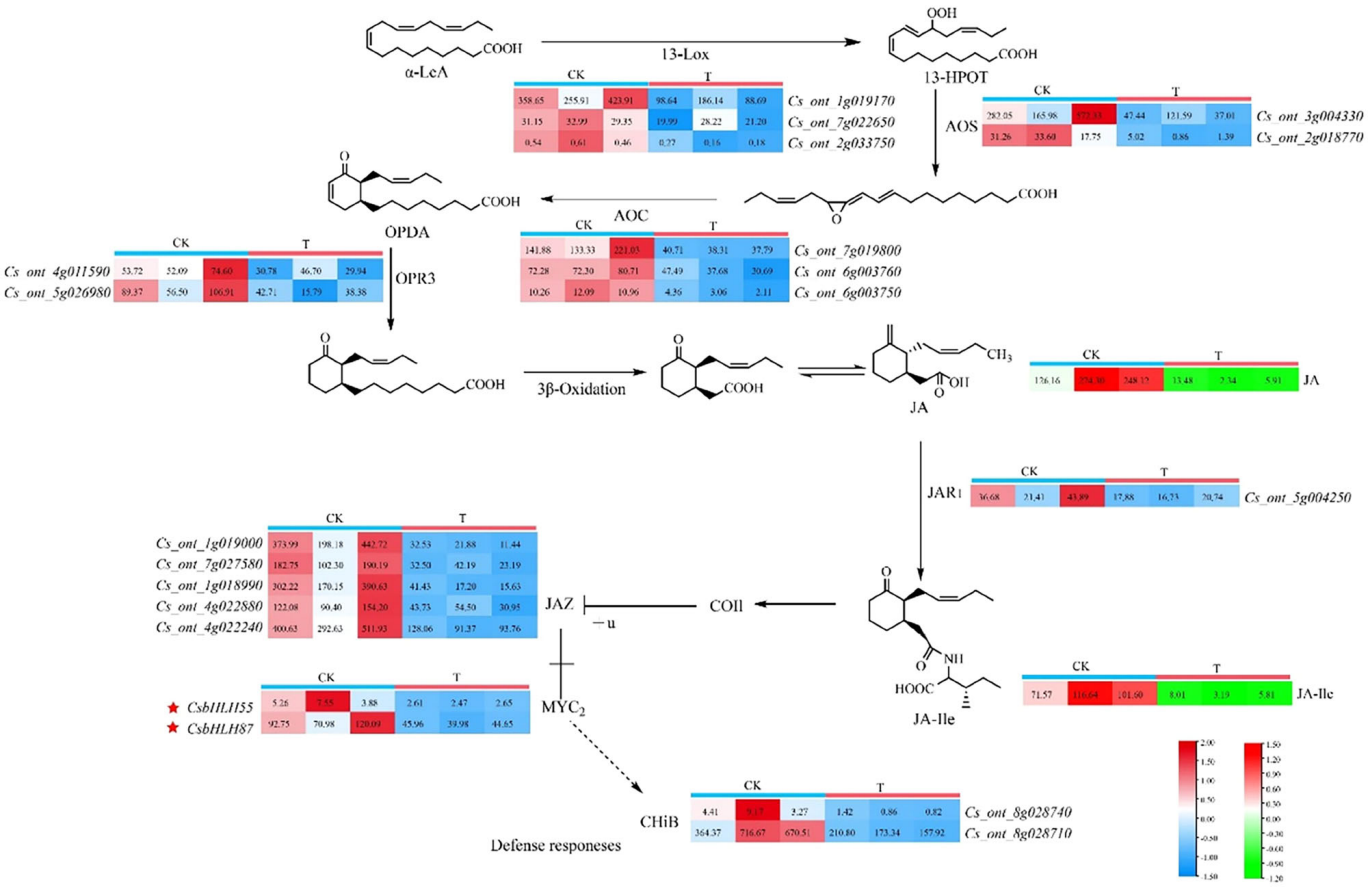
Signal transduction diagram of jasmonic acid (JA). The blue and red heatmaps in the figure indicate the expression of the relevant genes; the green and red heatmaps indicate the content of the hormones; CK indicates the control group cultured for 14 days in regular medium; and T indicates the treatment group cultured for 14 days in medium containing 2% NaCl. 13‐LOX, 13‐lipoxygenase; AOC, Allene oxide cyclase; AOS, Allene oxide synthase; OPR3, OPDA reductase; JAR1, jasmonate‐amino acid synthase gene; red pentagram: *bHLH* gene of sweet orange. The solid arrows in the figure indicate promotion, the dashed arrows indicate a series of processes in between, the vertical lines at the end of the solid lines indicate inhibition, and the solid lines with line segments in the middle indicate dissociation. JA‐Ile, jasmonic acid‐isoleucine.

In this study, we combined the results of homologous sequence alignment, transcriptome data, and targeted‐metabolome analysis to map DEGs and DAMs (differentially accumulated metabolites) from the same control group to KEGG pathway information to understand the relationships between genes and metabolites. Twenty candidate target genes for JA (Ko04075)‐related signaling pathways were identified (Figure 12), among which *CsbHLH55* and *CsbHLH87* in the *CsbHLH* family could be candidates for *MYC2* TFs. A total of 11 genes (three 13‐LOX, two AOS, three AOC, two OPR3, and one JAR1) may be involved in the synthesis of JA and JA‐Ile, and the expression of all 11 genes was downregulated under salt stress. The expression of genes involved in hormone synthesis, the content of synthesized hormones, and the expression of defense genes (CHiB) were downregulated, suggesting that the plant's salt tolerance decreased, which was consistent with the results of leaf curling and dropping after 14 days of salt stress (Figure 6A). However, whether the *bHLH55* and *bHLH87* genes are *MYC2* TFs and whether the remaining 18 genes are crucial in the JA signaling pathway requires further experimental evidence.

## 4 DISCUSSION

Sweet orange is one of the most widely grown fruit crops and has high economic value (Biswas et al., 2014). In recent years, the global trend of soil salinization has been exacerbated by anthropogenic breaching of the natural environment (Litalien & Zeeb, 2020). Salt stress, an abiotic stress, affects the development of the citrus industry worldwide, especially in arid and coastal areas (Bailey et al., 2003). Therefore, the selection of new varieties of sweet oranges that are tolerant to salt stress, the identification of salt tolerance genes, and the study of the mechanism of action of salt tolerance are crucial. *bHLH* genes play a role in phytohormone signaling and the response to biotic or abiotic stresses. Phytohormones regulate plant growth and development and play vital roles in biotic and abiotic stress responses. In this study, we identified the sweet orange *bHLH* gene family, analyzed the expression patterns of *CsbHLH* genes under salt stress, and analyzed the hormone content of sweet orange seedlings under salt stress, which can provide a reference for the cultivation of new salt‐resistant sweet orange varieties.

In this study, a total of 120 *bHLH* genes were identified in sweet orange, whereas 162 were identified in *A. thaliana* (Bailey et al., 2003), 167 in *O. sativa* (X. Li et al., 2006), 213 in *Raphanus sativus* (Wang et al., 2022), and 188 in *M. domestica* (Mao et al., 2017), suggesting that the number of *bHLH* genes is related to the genome size of different species. Phylogenetic analysis classified the *CsbHLH* genes into 18 subfamilies, consistent with the *bHLH* subfamilies of *A. thaliana*, and *CsbHLH* genes were identified in each subfamily of *A. thaliana*. These findings suggested that the *CsbHLH* genes may have performed some fundamental biological functions during the evolution of sweet orange. The expression of the *AtMYC2* (*AtbHLH6*) gene in *A. thaliana enhances salt* tolerance (J. Li et al., 2021), and *AtbHLH6* is a member of subfamily III with *CsbHLH55* and *CsbHLH87*, which are hypothesized to have similar functions.

Gene structure analysis revealed many variations in the number of introns in the sweet orange *bHLH* gene, with intron numbers ranging from 0 to 40. This gene is similar to the *bHLH* genes in *Rosa rugosa* (Ding et al., 2023), *Ginkgo biloba* (X. Zhou et al., 2020), and *Chimonanthus praecox* (Kamran et al., 2023). The number of introns in the *bHLH* genes of sweet orange varies widely, which can lead to diverse gene functions. In conclusion, the high similarity in gene structure and structural features of conserved motifs further suggested that the more similar the genes were during evolution, the more similar the functions were, thus facilitating the screening of bHLH genes with similar functions. For example, *AtbHLH112* in *A. thaliana* can respond to drought stress and salt stress by synthesizing proline and scavenging ROS (Y. Liu et al., 2015), the *OsbHLH006* and *OsbHLH148* genes in rice can respond to drought stress through the JA signaling pathway (Kiribuchi et al., 2004; Seo et al., 2011), and *TabHLH1* in tobacco can mediate the response of tobacco to osmotic stress (T. Yang et al., 2016). It can be hypothesized that *CsbHLH* genes play a crucial role in regulating abiotic stresses, including salt stress. The structural domains of the four gene sequences were predicted using the CD‐search tool to verify the accuracy of the ultralong sequences of *CsbHLH28*, *CsbHLH26*, *CsbHLH63*, and *CsbHLH99*, as shown in Figure 3. The results showed (Table S4) that all four bHLH gene sequences are similar and that there are similar ultralong bHLH genes among the bHLH genes of *Chimonanthus praecox* (Kamran et al., 2023).

Promoter cis‐acting elements play vital roles in regulating transcription and gene expression. Many resistance‐related cis‐acting elements were identified in the upstream 2000 bp sequence of the sweet orange *bHLH* gene. The cis‐acting elements in *CsbHLHs* are mainly classified into five categories, namely, phytohormone‐responsive elements, environmental stress‐responsive elements, light‐responsive elements, plant‐specific regulatory elements, and MYB‐binding sites, with the greatest proportion of phytohormone‐responsive elements. The phytohormone response elements in the *CsbZIP* genes were mainly ABA response elements, GA response elements, methyl jasmonate (MeJA) response elements, and salicylic acid (SA) response elements (Figure 4). Studies have shown that the bHLH TF family plays a crucial role in the regulation of secondary metabolism, such as the signaling of JA, ABA, GA, and SA (Aleman et al., 2016; Fukazawa et al., 2023; Gautam et al., 2021; M. Zhou & Memelink, 2016). Previous studies have shown that both MeJA and ABA can stimulate the expression of plant defense genes and induce chemical defenses and some physiological and functional stress responses in plants (Berens et al., 2017; S. Cao et al., 2013; Laura et al., 2018; Ma et al., 2018; Osakabe et al., 2014). The salt tolerance of plants can be affected by altering the level and signaling of GA (Colebrook et al., 2014); SA signaling can regulate plant seed germination, growth, flower induction, thermogenesis, and biotic and abiotic stress responses (Janda et al., 2020). bHLH TFs in sweet oranges are crucial for growth, development, and adversity stress regulation. GO enrichment analysis revealed hormone‐stimulated responses, such as those related to JA, GA, ABA, and JA‐mediated signaling pathways, and environmentally stimulated responses, such as responses to salt stress, hyperosmotic salinity, and other stress stimuli (Figure 8B; Table S5). The plant hormone signal transduction pathway and the MAPK signaling pathway were identified via KEGG enrichment analysis (Figure 7A; Table S9). These findings further suggested that CsbHLHSs can regulate plant growth and development by regulating hormone levels and signaling under adverse conditions.

Gene duplication is the primary mode of gene amplification and molecular mechanism for gene evolution to generate new functional genes (Yanhui et al., 2006). In this study, we analyzed gene duplication events in the *CsbHLH* gene family and detected 28 segmental duplication gene pairs among 120 *bHLH* genes (Figure 5A), indicating that these gene duplication events are also crucial for *CsbHLH* gene amplification. In addition, the greatest number of fragment duplication gene pairs occurred on chromosomes 2 and 5, and more *bHLH* genes were localized to chromosomes 2 and 5, suggesting that sweet orange chromosomes 2 and 5 have the potential to contain this relatively vital resource of *bHLH* genes.

Salt stress is one of the primary environmental stresses that limits plant growth and productivity (Yu et al., 2020). In this study, 30 sweet orange histocultures were divided into two groups of 15 plants each; one group was put into MS media supplemented with 2% NaCl, and the other was put into regular MS (Murashige and Skoog) media and cultured for 14 days. The leaves of sweet orange histocultures under salt stress turned yellow, curled, and even fell off; compared with those of the control group, the water content and fresh weight of the leaves significantly decreased, and their growth was poor (Figure 6). This change indicates that salt stress limits plant water and nutrient absorption (Gong, 2021) and inhibits photosynthesis to hinder plant growth, thus inhibiting cell division and expansion (Van et al., 2020).

Under environmental stress, plants can respond through corresponding life activities, during which DEGs may be involved in corresponding response pathways (Fan et al., 2022). Studying the expression patterns of related genes based on transcriptomic data is a new approach. In this study, we analyzed the expression patterns of sweet orange *bHLH* genes under salt stress. Seventeen out of the 120 *CsbHLH* genes were differentially and significantly expressed under salt stress; four were upregulated, and 13 were downregulated (Figure 7A). Three genes were selected from among the strongly downregulated genes and significantly upregulated genes for qRT‐PCR validation. The qRT‐PCR results strongly correlated with the transcriptome sequencing results, which confirmed the accuracy of the transcriptome data and verified the reliability of the transcriptome data concerning the differentially regulated genes (Figure 7B). These DEGs could respond to salt stress in sweet oranges. Based on the transcriptional data, the *CsbHLH55* and *CsbHLH87* genes could be candidates for *MYC2* TFs (Figure 12). MYC2, a subfamily of bHLH TFs, is a prime regulator of the *A. thaliana* response to JA, and JA can respond to salt stress through MYC2‐mediated expression of defense genes (Goossens et al., 2015; Song et al., 2021). Therefore, it is hypothesized that the *CsbHLH55* and *CsbHLH87* genes can also mediate JA signaling.

Phytohormones can mediate environmental stresses, including salt stress, and thus regulate plant growth (Yu et al., 2020). The hormone contents of sweet orange histocultured plants under salt stress were determined in the present study. The difference in the content of ABA was significant, as the difference in the content of JA was also significant compared to that in the control (Figure 9B). It has been shown that when plants are subjected to environmental stresses such as drought, salinity, and extreme temperatures, ABA accumulates to varying degrees (Min et al., 2021; X. Yang et al., 2022; A. Zhang et al., 2021). ABA can mediate abiotic stress through selective splicing. Therefore, plants under prolonged salt stress can be maintained by increasing ABA levels to induce defoliation.

Under various abiotic stresses, *bHLH* TFs play a vital role in gene regulation in many plant species (Jiang et al., 2019). It has been shown that the bHLH gene can encode *bHLH* either in an ABA‐dependent or ABA‐independent manner and regulate ABA signaling indirectly or directly (Hussain et al., 2021; Le et al., 2017). Under abiotic stress, ABA‐dependent or ABA‐independent bHLH genes inhibit ROS, reduce hydrogen peroxide (H_2_O_2_) content, increase proline hairiness, and increase the photosynthetic rate, thus improving plant tolerance (Figure S3). The exact CsBbHLH gene encoded by ABA identified in this study is unclear and needs to be further verified.

Under short‐term environmental stress, the endogenous JA content of plants increases transiently and rapidly but decreases with prolonged stress (Balbi & Devoto, 2008; Ollas et al., 2013; J. Wang et al., 2020). However, in the present study, the JA content decreased after 14 days of salt treatment, indicating that the 14‐day duration of salt stress was prolonged. Exogenous JA for 3 days reduces malondialdehyde (MDA) and H_2_O_2_ levels in wheat plants, significantly increasing the tolerance of those plants to salt stress (Qiu et al., 2014). Therefore, salt stress can be alleviated by the application of hormones such as exogenous JA, which stimulates JA signaling.

Phytohormones such as JA are regulators of plant development and participate in sensing and sending various environmental signals (Lymperopoulos et al., 2018). JA and its derivatives play vital roles in mediating the response and defense against abiotic stresses in plants (Y. Wang et al., 2021). Salt stress promotes the synthesis of α‐linolenic acid (α‐leA) in chloroplasts (Figure 12), which can be oxidized and converted to 12‐oxo‐phytodienoic acid (12‐oxo‐PDA) by 13‐lipoxygenase (13‐LOX), acryloxypropanolase (AOS), and acrylooxypropyl cyclase (AOC). Finally, 12‐oxo‐PDA is synthesized into JA via reduction by 12‐oxo‐PDA reductase (OPR3) and three cycles of β‐oxidation (Ruan et al., 2019; Thines et al., 2007). JA is coupled to isoleucine via JAR1 to form JA‐Ile (Fonseca et al., 2009; Staswick & Tiryaki, 2004). The transcriptional deterrent protein JAZ binds to the F‐box protein COI1 in a hormone‐dependent manner (Sheard et al., 2010). In the absence of hormone signals, the JAZ protein represses the *MYC2* TF, which binds to the cis‐acting element of JA‐responsive genes. JA stimulates the specific binding of the JAZ protein to COI1, which induces the degradation of JAZ. The degradation of JAZ removes the repression of the *MYC2* TF, which allows for the expression of JA‐responsive genes, thereby enhancing plant resistance (Chini et al., 2007; Lorenzo et al., 2004). *MYC2* is a major downstream effector in the JA signaling pathway, and JAZ is a regulator of JA signaling that binds to *MYC2*, thereby inhibiting the TF activity of MYC2 (Luo et al., 2023). JA negatively regulates plant salt stress tolerance by inhibiting the expression of specific genes in a MYC2‐dependent manner. *MYC2* can scavenge ROS through several enzymatic interactions, enhancing plant tolerance (Song et al., 2021).

**FIGURE 13:**
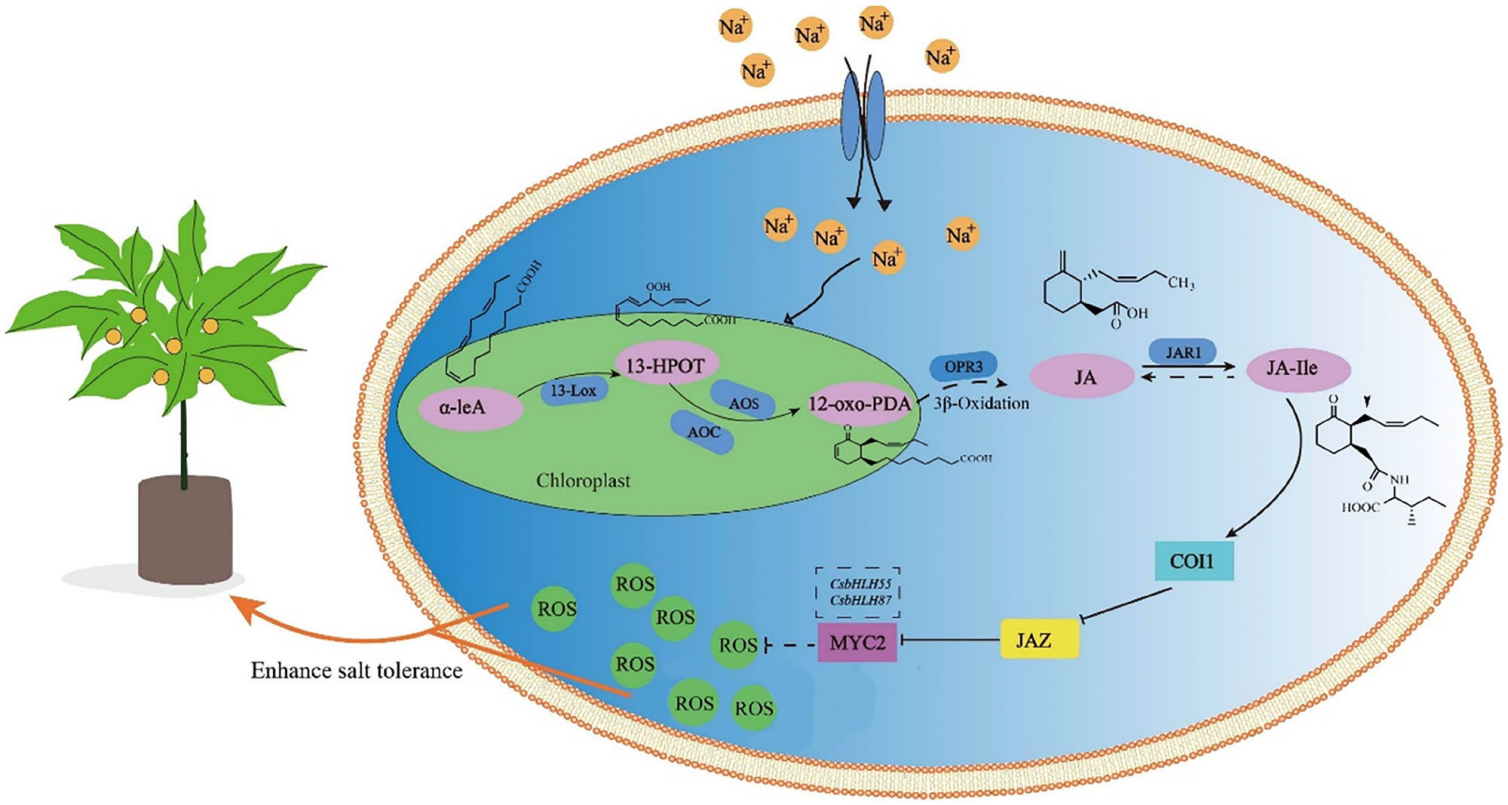
The mechanism of salt tolerance in sweet oranges involves *CsbHLH55* and *CsbHLH87* (members of the MYC2 subfamily). 12‐oxo‐PDA, 12‐oxo phytodienoic acid; 13‐HPOT, 13‐hydroperoxylated linolenic acid; 13‐LOX, 13‐lipoxygenase; AOC, epoxypropane cyclase; AOS, epoxypropenylase; COI1, F‐box CORONATINE INSENSITIVE 1; JA, jasmonic acid; JA‐Ile, jasmonic acid‐isoleucine; JAR1, jasmonate‐amino acid synthase; LOX, 13‐lipoxygenase; OPR3, 12‐oxo‐PDA reductase; α‐leA, α‐linolenic acid.

*MYC2* is a *bHLH* TF that acts as a regulatory hub in several signaling pathways (Verma et al., 2020). MYC proteins belong to the *bHLH* family and contain the specific structural domain JID (Fernández‐Calvo et al., 2011). In this study, the *CsbHLH55* and *CsbHLH87* genes were identified as candidate genes for the *MYC2* TF in sweet orange by sequence alignment, protein three‐dimensional structure prediction (Figure 11), KEGG enrichment analysis (Figure 10), and combined analysis of transcriptome and metabolome data (Figure 12). Studies have shown that overexpression of the *MYC2* gene enhances stress resistance (Deng et al., 2023; Wang et al., 2022). In summary, multiple lines of evidence suggest that sweet orange *bHLH* genes play a vital role in sweet orange resistance to abiotic stresses such as salt stress and that the *CsbHLH55* and *CsbHLH87* genes can be used as candidate genes for the breeding of new transgenic salt‐resistant varieties of sweet orange. The expression of *CsbHLH55* and *CsbHLH87*, candidate genes for sweet orange *MYC2* TFs, which are major downstream effectors of the JA signaling pathway, can increase salt tolerance, which is the mechanism of *bHLH* involvement in salt stress tolerance that distinguishes sweet orange plants from other plants (Figure 13).

## 5 CONCLUSION

In this study, 120 *CsbHLH* genes were identified from the sweet orange genome and were phylogenetically classified into 18 subfamilies. The growth of the sweet orange group of plants was strongly inhibited when the plants were cultured in media supplemented with 2% NaCl for 14 days. Transcriptome sequencing revealed that all 120 *CsbHLH* genes were expressed under salt stress, and 17 were DEGs. The qRT‐PCR results showed a strong correlation with the transcriptome data, which confirmed the accuracy of the transcriptome data. The results of metabolite measurements showed that the difference in ABA content was significantly upregulated, and the difference in JA and JA‐Ile content was significantly downregulated, suggesting that phytohormones play a crucial role in resistance to salt stress. Twenty genes differentially expressed during JA signaling served as target genes, among which *CsbHLH55* and *CsbHLH87* of the sweet orange CsbHLH gene family were identified as target genes of the *MYC2* TF, which can be activated and involved in the JA signaling pathway. Activation of the JA signaling pathway inhibits the production of ROS, which in turn improves the salt tolerance of sweet orange plants, and it is a vital mechanism through which sweet orange *bHLH* genes participate in the regulation of salt stress tolerance.

## AUTHOR CONTRIBUTIONS

**Yinqiang Zi**: Writing—original draft; writing—review and editing. **Mengjie Zhang**: Data curation; visualization; writing—review and editing. **Xiuyao Yang**: Supervision; validation. **Ke Zhao**: Software; validation. **Tuo Yin**: Methodology. **Ke Wen**: Formal analysis; methodology. **Xulin Li**: Investigation; software. **Xiaozhen Liu**: Project administration; supervision. **Hanyao Zhang**: Funding acquisition; supervision.

## CONFLICT OF INTEREST STATEMENT

The authors declare no conflicts of interest.

## Supporting information

Supplementary Figures: Figure S1, Correlation coefficient plot of qRT‐PCR results with FPKM values of transcriptome data. Different colors indicate the correlation coefficients of the qRT‐PCR results of various genes with the FPKM values of the transcriptome data.
Supplementary Tables: Table S1, Gene IDs and names of *Arabidopsis thaliana* bHLH genes; Table S2, Heatmap of expression of six differentially expressed genes; Table S2, Design of qRT‐PCR primers for eight CsbHLH genes in sweet orange; Table S3, Analysis of physicochemical properties of CsbHLH TFs; Table S4, Structural domain prediction of CsbHLH28, 26, 63 and 99 genes; Table S5, GO enrichment analysis of sweet orange bHLH genes; Table S6, Physiological indices of sweet orange seedlings analyzed under salt stress; Table S7, FPKM values of bHLH differentially significant genes in sweet orange under salt stress; Table S8, Raw CT values of qRT‐PCR; Table S9, KEGG enrichment analysis of CsbHLH genes under salt stress; Table S10, Changes in phytohormone content under salt stress (14 days).

## Data Availability

All the data generated or analyzed during this study are included in this published article. The *C. sinensis* genome (v3.0) and annotation files of sweet orange are openly available in the CPBD: Citrus Pangenome to Breeding Database (https://citrus.hzau.edu.cn/download.php). RNA‐Seq data under salt stress conditions can be found under the accession numbers GSE248845, GSM7921250, GSM7921251, GSM7921252, GSM7921253, and GSM7921255. The RNA‐Seq data are publicly available at the National Center for Biotechnology Information. The other data presented in this study are available in the Supplementary Materials.

## References

[tpg220502-bib-0001] Aleman, F. , Yazaki, J. , Lee, M. , Takahashi, Y. , Kim, A. Y. , Li, Z. , Kinoshita, T. , Ecker, J. R. , & Schroeder, J. I. (2016). An ABA‐increased interaction of the PYL6 ABA receptor with MYC2 transcription factor: A putative link of ABA and JA signaling. Scientific Reports, 6, 28941. 10.1038/srep28941 27357749 PMC4928087

[tpg220502-bib-0002] Arif, Y. , Singh, P. , Siddiqui, H. , Bajguz, A. , & Hayat, S. (2020). Salinity induced physiological and biochemical changes in plants: An omic approach towards salt stress tolerance. Plant Physiology and Biochemistry, 156, 64–77. 10.1016/j.plaphy.2020.08.042 32906023

[tpg220502-bib-0003] Bailey, P. C. , Martin, C. , Toledo‐Ortiz, G. , Quail, P. H. , Huq, E. , Heim, M. A. , Jakoby, M. , Werber, M. , & Weisshaar, B. (2003). Update on the basic helix‐loop‐helix transcription factor gene family in *Arabidopsis thaliana* . The Plant Cell, 15(11), 2497–2502. 10.1105/tpc.151140 14600211 PMC540267

[tpg220502-bib-0004] Balbi, V. , & Devoto, A. (2008). Jasmonate signalling network in *Arabidopsis thaliana*: Crucial regulatory nodes and new physiological scenarios. New Phytologist, 177(2), 301–318. 10.1111/j.1469-8137.2007.02292.x 18042205

[tpg220502-bib-0005] Berens, M. L. , Berry, H. M. , Mine, A. , Argueso, C. T. , & Tsuda, K. (2017). Evolution of hormone signaling networks in plant defense. Annual Review of Phytopathology, 55, 401–425. 10.1146/annurev-phyto-080516-035544 28645231

[tpg220502-bib-0006] Biswas, M. K. , Xu, Q. , Mayer, C. , & Deng, X. (2014). Genome wide characterization of short tandem repeat markers in sweet orange (*Citrus sinensis*). PLoS One, 9(8), e104182. 10.1371/journal.pone.0104182 25148383 PMC4141690

[tpg220502-bib-0007] Cao, S. , Cai, Y. , Yang, Z. , Joyce, D. C. , & Zheng, Y. (2013). Effect of MeJA treatment on polyamine, energy status and anthracnose rot of loquat fruit. Food Chemistry, 145, 86–89. 10.1016/j.foodchem.2013.08.019 24128452

[tpg220502-bib-0008] Cao, Y. , Liu, L. , Ma, K. , Wang, W. , Lv, H. , Gao, M. , Wang, X. , Zhang, X. , Ren, S. , Zhang, N. , & Guo, Y. D. (2022). The jasmonate‐induced *bHLH* gene *SlJIG* functions in terpene biosynthesis and resistance to insects and fungus. Journal of Integrative Plant Biology, 64(5), 1102–1115. 10.1111/jipb.13248 35293128

[tpg220502-bib-0009] Chen, C. , Chen, H. , Zhang, Y. , Thomas, H. R. , Frank, M. H. , He, Y. , & Xia, R. (2020). TBtools: An integrative toolkit developed for interactive analyses of big biological data. Molecular Plant, 13(8), 1194–1202. 10.1016/j.molp.2020.06.009 32585190

[tpg220502-bib-0010] Chini, A. , Fonseca, S. , Fernández, G. , Adie, B. , Chico, J. M. , Lorenzo, O. , García‐Casado, G. , López‐Vidriero, I. , Lozano, F. M. , Ponce, M. R. , Micol, J. L. , & Solano, R. (2007). The JAZ family of repressors is the missing link in jasmonate signalling. Nature, 448(7154), 666–671. 10.1038/nature06006 17637675

[tpg220502-bib-0011] Colebrook, E. H. , Thomas, S. G. , Phillips, A. L. , & Hedden, P. (2014). The role of gibberellin signalling in plant responses to abiotic stress. Journal of Experimental Biology, 217(Pt 1), 67–75. 10.1242/jeb.089938 24353205

[tpg220502-bib-0012] De, A. B. , Rodrigo, M. J. , Sánchez‐Moreno, C. , Pilar, C. M. , & Zacarías, L. (2020). Effect of high‐pressure processing applied as pretreatment on carotenoids, flavonoids and vitamin C in juice of the sweet oranges ‘Navel’ and the red‐fleshed ‘Cara Cara’. Food Research International, 132, 109105. 10.1016/j.foodres.2020.109105 32331674

[tpg220502-bib-0013] Deng, H. , Li, Q. , Cao, R. , Ren, Y. , Wang, G. , Guo, H. , Bu, S. , Liu, J. , & Ma, P. (2023). Overexpression of *SmMYC2* enhances salt resistance in *Arabidopsis thaliana* and *Salvia miltiorrhiza* hairy roots. Journal of Plant Physiology, 280, 153862. 10.1016/j.jplph.2022.153862 36399834

[tpg220502-bib-0014] Ding, C. , Gao, J. , Zhang, S. , Jiang, N. , Su, D. , Huang, X. , & Zhang, Z. (2023). The basic/helix‐loop‐helix transcription factor family gene *RcbHLH112* is a susceptibility gene in gray mould resistance of rose (*Rosa chinensis*). International Journal of Molecular Sciences, 24(22), 16305. 10.3390/ijms242216305 38003495 PMC10671410

[tpg220502-bib-0015] Duan, S. , Xu, Z. , Li, X. Y. , Liao, P. , Qin, H. K. , Mao, Y. P. , Dai, W. S. , Ma, H. J. , & Bao, M. L. (2022). Dodder‐transmitted mobile systemic signals activate a salt‐stress response characterized by a transcriptome change in *Citrus sinensis* . Frontiers in Plant Science, 13, 986365. 10.3389/fpls.2022.986365 36046588 PMC9422749

[tpg220502-bib-0016] Fan, G. , Xia, X. , Yao, W. , Cheng, Z. , Zhang, X. , Jiang, J. , Zhou, B. , & Jiang, T. (2022). Genome‐wide identification and expression patterns of the F‐box family in poplar under salt stress. International Journal of Molecular Sciences, 23(18), 10934. 10.3390/ijms231810934 36142847 PMC9505895

[tpg220502-bib-0017] Fang, S. , Hou, X. , & Liang, X. (2021). Response mechanisms of plants under saline‐alkali stress. Frontiers in Plant Science, 12, 667458. 10.3389/fpls.2021.667458 34149764 PMC8213028

[tpg220502-bib-0018] Fernández‐Calvo, P. , Chini, A. , Fernández‐Barbero, G. , Chico, J. M. , Gimenez‐Ibanez, S. , Geerinck, J. , Eeckhout, D. , Schweizer, F. , Godoy, M. , Franco‐Zorrilla, J. M. , Pauwels, L. , Witters, E. , Puga, M. I. , Paz‐Ares, J. , Goossens, A. , Reymond, P. , De Jaeger, G. , & Solano, R. (2011). The *Arabidopsis* bHLH transcription factors MYC3 and MYC4 are targets of JAZ repressors and act additively with MYC2 in the activation of jasmonate responses. The Plant Cell, 23(2), 701–715. 10.1105/tpc.110.080788 21335373 PMC3077776

[tpg220502-bib-0019] Fonseca, S. , Chini, A. , Hamberg, M. , Adie, B. , Porzel, A. , Kramell, R. , Miersch, O. , Wasternack, C. , & Solano, R. (2009). (+)‐7‐*iso*‐Jasmonoyl‐L‐isoleucine is the endogenous bioactive jasmonate. Nature Chemical Biology, 5(5), 344–350. 10.1038/nchembio.161 19349968

[tpg220502-bib-0020] Fukazawa, J. , Mori, K. , Ando, H. , Mori, R. , Kanno, Y. , Seo, M. , & Takahashi, Y. (2023). Jasmonate inhibits plant growth and reduces gibberellin levels via microRNA5998 and transcription factor MYC2. Plant Physiology, 193(3), 2197–2214. 10.1093/plphys/kiad453 37562026

[tpg220502-bib-0021] Gautam, J. K. , Giri, M. K. , & Singh, D. , Chattopadhyay, S. , & Nandi, A. K. (2021). MYC2 influences salicylic acid biosynthesis and defense against bacterial pathogens in *Arabidopsis thaliana* . Physiologia Plantarum, 173(4), 2248–2261. 10.1111/ppl.13575 34596247

[tpg220502-bib-0022] Gong, Z. (2021). Plant abiotic stress: New insights into the factors that activate and modulate plant responses. Journal of Integrative Plant Biology, 63(3), 429–430. 10.1111/jipb.13079 33657281

[tpg220502-bib-0023] Goossens, J. , Swinnen, G. , Vanden, B. R. , Pauwels, L. , & Goossens, A. (2015). Change of a conserved amino acid in the MYC2 and MYC3 transcription factors leads to release of JAZ repression and increased activity. New Phytologist, 206(4), 1229–1237. 10.1111/nph.13398 25817565

[tpg220502-bib-0024] Guo, J. , Sun, B. , He, H. , Zhang, Y. , Tian, H. , & Wang, B. (2021). Current understanding of *bHLH* transcription factors in plant abiotic stress tolerance. International Journal of Molecular Sciences, 22(9), 4921. 10.3390/ijms22094921 34066424 PMC8125693

[tpg220502-bib-0025] He, X. , Zhang, W. , Sabir, I. A. , Jiao, C. , Li, G. , Wang, Y. , Zhu, F. , Dai, J. , Liu, L. , Chen, C. , Zhang, Y. , & Song, C. (2023). The spatiotemporal profile of *Dendrobium huoshanense* and functional identification of *bHLH* genes under exogenous MeJA using comparative transcriptomics and genomics. Frontiers in Plant Science, 14, 1169386. 10.3389/fpls.2023.1169386 37235024 PMC10206334

[tpg220502-bib-0026] Hou, X. , Singh, S. K. , Werkman, J. R. , Liu, Y. , Yuan, Q. , Wu, X. , Patra, B. , Sui, X. , Lyu, R. , Wang, B. , Liu, X. , Li, Y. , Ma, W. , Pattanaik, S. , & Yuan, L. (2023). Partial desensitization of MYC2 transcription factor alters the interaction with jasmonate signaling components and affects specialized metabolism. International Journal of Biological Macromolecules, 252, 126472. 10.1016/j.ijbiomac.2023.126472 37625752

[tpg220502-bib-0027] Huang, X. , Wang, Y. , & Wang, N. (2022). Highly efficient generation of canker‐resistant sweet orange enabled by an improved CRISPR/Cas9 system. Frontiers in Plant Science, 12, 769907. 10.3389/fpls.2021.769907 35087548 PMC8787272

[tpg220502-bib-0028] Hussain, Q. , Asim, M. , Zhang, R. , Khan, R. , Farooq, S. , & Wu, J. (2021). Transcription factors interact with ABA through gene expression and signaling pathways to mitigate drought and salinity stress. Biomolecules, 211(8), 1159. 10.3390/biom11081159 PMC8393639 34439825

[tpg220502-bib-0029] Jagodzik, P. , Tajdel‐Zielinska, M. , Ciesla, A. , Marczak, M. , & Ludwikow, A. (2018). Mitogen‐activated protein kinase cascades in plant hormone signaling. Frontiers in Plant Science, 9, 1387. 10.3389/fpls.2018.01387 30349547 PMC6187979

[tpg220502-bib-0030] Janda, T. , Szalai, G. , & Pál, M. (2020). Salicylic acid signalling in plants. International Journal of Molecular Sciences, 21(7), 2655. 10.3390/ijms21072655 32290350 PMC7177609

[tpg220502-bib-0031] Jiang, L. , Tian, X. , Li, S. , Fu, Y. , Xu, J. , & Wang, G. (2019). The *AabHLH35* transcription factor identified from *Anthurium andraeanum* is involved in cold and drought tolerance. Plants, 8(7), 216. 10.3390/plants8070216 31373334 PMC6681207

[tpg220502-bib-0032] Kamran, H. M. , Fu, X. , Wang, H. , Yang, N. , & Chen, L. (2023). Genome‐wide identification and expression analysis of the *bHLH* transcription factor family in wintersweet (*Chimonanthus praecox*). International Journal of Molecular Sciences, 24(17), 13462. 10.3390/ijms241713462 37686265 PMC10487621

[tpg220502-bib-0033] Katoh, K. , & Standley, D. M. (2013). MAFFT multiple sequence alignment software version 7: Improvements in performance and usability. Molecular Biology and Evolution, 30(4), 772–780. 10.1093/molbev/mst010 23329690 PMC3603318

[tpg220502-bib-0034] Kearse, M. , Moir, R. , Wilson, A. , Stones‐Havas, S. , Cheung, M. , Sturrock, S. , Buxton, S. , Cooper, A. , Markowitz, S. , Duran, C. , Thierer, T. , Ashton, B. , Meintjes, P. , & Drummond, A. (2012). Geneious basic: An integrated and extendable desktop software platform for the organization and analysis of sequence data. Bioinformatics, 28(12), 1647–1649. 10.1093/bioinformatics/bts199 22543367 PMC3371832

[tpg220502-bib-0035] Kiribuchi, K. , Sugimori, M. , Takeda, M. , Otani, T. , Okada, K. , Onodera, H. , Ugaki, M. , Tanaka, Y. , Tomiyama‐Akimoto, C. , Yamaguchi, T. , Minami, E. , Shibuya, N. , Omori, T. , Nishiyama, M. , Nojiri, H. , & Yamane, H. (2004). *RERJ1*, a jasmonic acid‐responsive gene from rice, encodes a basic helix‐loop‐helix protein. Biochemical and Biophysical Research Communications, 325(3), 857–863. 10.1016/j.bbrc.2004.10.126 15541369

[tpg220502-bib-0036] Laura, B. , Silvia, P. , Francesca, F. , Benedetta, S. , & Carla, C. (2018). Epigenetic control of defense genes following MeJA‐induced priming in rice (*O. sativa*). Journal of Plant Physiology, 228, 166–177. 10.1016/j.jplph.2018.06.007 29936261

[tpg220502-bib-0037] Le, H. R. , Castelain, M. , Chakraborti, D. , Moritz, T. , Dinant, S. , & Bellini, C. (2017). At*bHLH68* transcription factor contributes to the regulation of ABA homeostasis and drought stress tolerance in *Arabidopsis thaliana* . Physiologia Plantarum, 160, 312–327. 10.1111/ppl.12549 28369972

[tpg220502-bib-0038] Li, J. , Chen, S. , Yin, Y. , Shan, Q. , Zheng, C. , & Chen, Y. (2023). Genome‐wide analysis of *bHLH* family genes and identification of members associated with cold/drought‐induced photoinhibition in *Kandelia obovata* . International Journal of Molecular Sciences, 24(21), 15942. 10.3390/ijms242115942 37958925 PMC10647802

[tpg220502-bib-0039] Li, J. , Li, X. , Han, P. , Liu, H. , Gong, J. , Zhou, W. , Shi, B. , Liu, A. , & Xu, L. (2021). Genome‐wide investigation of *bHLH* genes and expression analysis under different biotic and abiotic stresses in *Helianthus annuus* L. International Journal of Biological Macromolecules, 189, 72–83. 10.1016/j.ijbiomac.2021.08.072 34411617

[tpg220502-bib-0040] Li, J. , Wang, T. , Han, J. , & Ren, Z. (2020). Genome‐wide identification and characterization of cucumber *bHLH* family genes and the functional characterization of *CsbHLH041* in NaCl and ABA tolerance in *Arabidopsis* and cucumber. BMC Plant Biology, 20(1), Article 272. 10.1186/s12870-020-02440-1 32527214 PMC7291561

[tpg220502-bib-0041] Li, X. , Duan, X. , Jiang, H. , Sun, Y. , Tang, Y. , Yuan, Z. , Guo, J. , Liang, W. , Chen, L. , Yin, J. , Ma, H. , Wang, J. , & Zhang, D. (2006). Genome‐wide analysis of basic/helix‐loop‐helix transcription factor family in rice and *Arabidopsis* . Plant Physiology, 141(4), 1167–1184. 10.1104/pp.106.080580 16896230 PMC1533929

[tpg220502-bib-0042] Litalien, A. , & Zeeb, B. (2020). Curing the earth: A review of anthropogenic soil salinization and plant‐based strategies for sustainable mitigation. Science of the Total Environment, 698, 134235. 10.1016/j.scitotenv.2019.134235 31783465

[tpg220502-bib-0043] Liu, W. , Tai, H. , Li, S. , Gao, W. , Zhao, M. , Xie, C. , & Li, W. X. (2014). *bHLH122* is important for drought and osmotic stress resistance in *Arabidopsi*s and in the repression of ABA catabolism. New Phytologist, 201(4), 1192–1204. 10.1111/nph.12607 24261563

[tpg220502-bib-0044] Liu, Y. , Ji, X. , Nie, X. , Qu, M. , Zheng, L. , Tan, Z. , Zhao, H. , Huo, L. , Liu, S. , Zhang, B. , & Wang, Y. (2015). *Arabidopsis AtbHLH112* regulates the expression of genes involved in abiotic stress tolerance by binding to their E‐box and GCG‐box motifs. New Phytologist, 207(3), 692–709. 10.1111/nph.13387 25827016

[tpg220502-bib-0045] Lorenzo, O. , Chico, J. M. , Sánchez‐Serrano, J. J. , & Solano, R. (2004). *JASMONATE‐INSENSITIVE1* encodes a MYC transcription factor essential to discriminate between different jasmonate‐regulated defense responses in *Arabidopsis* . The Plant Cell, 16(7), 1938–1950. 10.1105/tpc.022319 15208388 PMC514172

[tpg220502-bib-0046] Luo, L. , Wang, Y. , Qiu, L. , Han, X. , Zhu, Y. , Liu, L. , Man, M. , Li, F. , Ren, M. , & Xing, Y. (2023). MYC2: A master switch for plant physiological processes and specialized metabolite synthesis. International Journal of Molecular Sciences, 24(4), 3511. 10.3390/ijms24043511 36834921 PMC9963318

[tpg220502-bib-0047] Lymperopoulos, P. , Msanne, J. , & Rabara, R. (2018). Phytochrome and phytohormones: Working in tandem for plant growth and development. Frontiers in Plant Science, 9, 1037. 10.3389/fpls.2018.01037 30100912 PMC6072860

[tpg220502-bib-0048] Ma, Y. , Cao, J. , He, J. , Chen, Q. , Li, X. , & Yang, Y. (2018). Molecular mechanism for the regulation of ABA homeostasis during plant development and stress responses. International Journal of Molecular Sciences, 19(11), 3643. 10.3390/ijms19113643 30463231 PMC6274696

[tpg220502-bib-0049] Mahmoud, L. M. , Huyck, P. J. , Vincent, C. I. , Gmitter, F. G., Jr. , Grosser, J. W. , & Dutt, M. (2021). Physiological responses and gene expression patterns in open‐pollinated seedlings of a pummelo‐mandarin hybrid rootstock exposed to salt stress and huanglongbing. Plants, 10(7), 1439. 10.3390/plants10071439 34371641 PMC8309399

[tpg220502-bib-0050] Mao, K. , Dong, Q. , Li, C. , Liu, C. , & Ma, F. (2017). Genome wide identification and characterization of apple *bHLH* transcription factors and expression analysis in response to drought and salt stress. Frontiers in Plant Science, 8, 480. 10.3389/fpls.2017.00480 28443104 PMC5387082

[tpg220502-bib-0051] Min, M. K. , Kim, R. , Hong, W. J. , Jung, K. H. , Lee, J. Y. , & Kim, B. G. (2021). OsPP2C09 is a bifunctional regulator in both ABA‐dependent and Independent abiotic stress signaling pathways. International Journal of Molecular Sciences, 22(1), 393. 10.3390/ijms22010393 33401385 PMC7795834

[tpg220502-bib-0052] Niu, X. , & Fu, D. (2022). The roles of BLH transcription factors in plant development and environmental response. International Journal of Molecular Sciences, 23(7), 3731. 10.3390/ijms23073731 35409091 PMC8998993

[tpg220502-bib-0053] Ollas, C. , Hernando, B. , Arbona, V. , & Gómez‐Cadenas, A. (2013). Jasmonic acid transient accumulation is needed for abscisic acid increase in citrus roots under drought stress conditions. Physiologia Plantarum, 147(3), 296–306. 10.1111/j.1399-3054.2012.01659.x 22671923

[tpg220502-bib-0054] Osakabe, Y. , Yamaguchi‐Shinozaki, K. , Shinozaki, K. , & Tran, L. P. (2014). ABA control of plant macroelement membrane transport systems in response to water deficit and high salinity. New Phytologist, 202(1), 35–49. 10.1111/nph.12613 24283512

[tpg220502-bib-0055] Peñuelas, M. , Monte, I. , Schweizer, F. , Vallat, A. , Reymond, P. , García‐Casado, G. , Franco‐Zorrilla, J. M. , & Solano, R. (2019). Jasmonate‐related MYC transcription factors are functionally conserved in *Marchantia polymorpha* . The Plant Cell, 31(10), 2491–2509. 10.1105/tpc.18.00974 31391256 PMC6790078

[tpg220502-bib-0056] Potter, S. C. , Luciani, A. , Eddy, S. R. , Park, Y. , Lopez, R. , & Finn, R. D. (2018). HMMER web server: 2018 update. Nucleic Acids Research, 46(W1), W200–W204. 10.1093/nar/gky448 29905871 PMC6030962

[tpg220502-bib-0057] Qiu, Z. , Guo, J. , Zhu, A. , Zhang, L. , & Zhang, M. (2014). Exogenous jasmonic acid can enhance tolerance of wheat seedlings to salt stress. Ecotoxicology and Environmental Safety, 104, 202–208. 10.1016/j.ecoenv.2014.03.014 24726929

[tpg220502-bib-0058] Ruan, J. , Zhou, Y. , Zhou, M. , Yan, J. , Khurshid, M. , Weng, W. , Cheng, J. , & Zhang, K. (2019). Jasmonic acid signaling pathway in plants. International Journal of Molecular Sciences, 20(10), 2479. 10.3390/ijms20102479 31137463 PMC6566436

[tpg220502-bib-0059] Seo, J. S. , Joo, J. , Kim, M. J. , Kim, Y. K. , Nahm, B. H. , Song, S. I. , Cheong, J. J. , Lee, J. S. , Kim, J. K. , & Choi, Y. D. (2011). *OsbHLH148*, a basic helix‐loop‐helix protein, interacts with *OsJAZ* proteins in a jasmonate signaling pathway leading to drought tolerance in rice. Plant Journal, 65(6), 907–921. 10.1111/j.1365-313X.2010.04477.x 21332845

[tpg220502-bib-0060] Sheard, L. B. , Tan, X. , Mao, H. , Withers, J. , Ben‐Nissan, G. , Hinds, T. R. , Kobayashi, Y. , Hsu, F. F. , Sharon, M. , Browse, J. , He, S. Y. , Rizo, J. , Howe, G. A. , & Zheng, N. (2010). Jasmonate perception by inositol‐phosphate‐potentiated COI1‐JAZ co‐receptor. Nature, 468(7322), 400–405. 10.1038/nature09430 20927106 PMC2988090

[tpg220502-bib-0061] Song, R. F. , Li, T. T. , & Liu, W. C. (2021). Jasmonic acid impairs *Arabidopsis* seedling salt stress tolerance through MYC2‐mediated repression of *CAT2* expression. Frontiers in Plant Science, 12, 730228. 10.3389/fpls.2021.730228 34745163 PMC8569249

[tpg220502-bib-0062] Staswick, P. E. , & Tiryaki, I. (2004). The oxylipin signal jasmonic acid is activated by an enzyme that conjugates it to isoleucine in *Arabidopsis* . The Plant Cell, 16(8), 2117–2127. 10.1105/tpc.104.023549 15258265 PMC519202

[tpg220502-bib-0063] Sun, H. , Fan, H. J. , & Ling, H. Q. (2015). Genome‐wide identification and characterization of the *bHLH* gene family in tomato. BMC Genomics, 16(1), Article 9. 10.1186/s12864-014-1209-2 25612924 PMC4312455

[tpg220502-bib-0064] Sun, J. , He, L. , & Li, T. (2019). Response of seedling growth and physiology of *Sorghum bicolor* (L.) Moench to saline‐alkali stress. PLoS One, 14(7), e0220340. 10.1371/journal.pone.0220340 31361760 PMC6667135

[tpg220502-bib-0065] Sun, X. , Wang, Y. , & Sui, N. (2018). Transcriptional regulation of *bHLH* during plant response to stress. Biochemical and Biophysical Research Communications, 503(2), 397–401. 10.1016/j.bbrc.2018.07.123 30057319

[tpg220502-bib-0066] Thines, B. , Katsir, L. , Melotto, M. , Niu, Y. , Mandaokar, A. , Liu, G. , Nomura, K. , He, S. Y. , Howe, G. A. , & Browse, J. (2007). JAZ repressor proteins are targets of the SCF^COI1^ complex during jasmonate signalling. Nature, 448(7154), 661–665. 10.1038/nature05960 17637677

[tpg220502-bib-0067] Van, Z. E. , Zhang, Y. , & Testerink, C. (2020). Salt tolerance mechanisms of plants. Annual Review of Plant Biology, 71, 403–433. 10.1146/annurev-arplant-050718-100005 32167791

[tpg220502-bib-0068] Verma, D. , Jalmi, S. K. , Bhagat, P. K. , Verma, N. , & Sinha, A. K. (2020). A bHLH transcription factor, MYC2, imparts salt intolerance by regulating proline biosynthesis in *Arabidopsis* . The FEBS Journal, 287(12), 2560–2576. 10.1111/febs.15157 31782895

[tpg220502-bib-0069] Verma, V. , Ravindran, P. , & Kumar, P. P. (2016). Plant hormone‐mediated regulation of stress responses. BMC Plant Biology, 16, Article 86. 10.1186/s12870-016-0771-y 27079791 PMC4831116

[tpg220502-bib-0070] Wang, J. , Song, L. , Gong, X. , Xu, J. , & Li, M. (2020). Functions of jasmonic acid in plant regulation and response to abiotic stress. International Journal of Molecular Sciences, 21(4), 1446. 10.3390/ijms21041446 32093336 PMC7073113

[tpg220502-bib-0071] Wang, L. , Xiang, L. , Hong, J. , Xie, Z. , & Li, B. (2019). Genome‐wide analysis of *bHLH* transcription factor family reveals their involvement in biotic and abiotic stress responses in wheat (*Triticum aestivum* L.). 3 Biotech, 9(6), Article 236. 10.1007/s13205-019-1742-4 PMC6536565 31139551

[tpg220502-bib-0072] Wang, P. , Su, L. , Gao, H. , Jiang, X. , Wu, X. , Li, Y. , Zhang, Q. , Wang, Y. , & Ren, F. (2018). Genome‐wide characterization of *bHLH g*enes in grape and analysis of their potential relevance to abiotic stress tolerance and secondary metabolite biosynthesis. Frontiers in Plant Science, 9, 64. 10.3389/fpls.2018.00064 29449854 PMC5799661

[tpg220502-bib-0074] Wang, R. , Yu, M. , Xia, J. , Xing, J. , Fan, X. , Xu, Q. , Cang, J. , & Zhang, D. (2022). Overexpression of *TaMYC2* confers freeze tolerance by ICE‐CBF‐COR module in *Arabidopsis thaliana* . Frontiers in Plant Science, 13, 1042889. 10.3389/fpls.2022.1042889 36466238 PMC9710523

[tpg220502-bib-0075] Wang, Y. , Mostafa, S. , Zeng, W. , & Jin, B. (2021). Function and mechanism of jasmonic acid in plant responses to abiotic and biotic stresses. International Journal of Molecular Sciences, 22(16), 8568. 10.3390/ijms22168568 34445272 PMC8395333

[tpg220502-bib-0076] Waseem, M. , Rong, X. , & Li, Z. (2019). Dissecting the role of a basic helix‐loop‐helix transcription factor, *SlbHLH22*, under salt and drought stresses in transgenic *Solanum lycopersicum* L. Frontiers in Plant Science, 10, 734. 10.3389/fpls.2019.00734 31231412 PMC6558761

[tpg220502-bib-0077] Wasternack, C. , & Hause, B. (2013). Jasmonates: Biosynthesis, perception, signal transduction and action in plant stress response, growth and development. An update to the 2007 review in Annals of Botany. Annals of Botany, 111(6), 1021–1058. 10.1093/aob/mct067 23558912 PMC3662512

[tpg220502-bib-0078] Waterhouse, A. M. , Procter, J. B. , Martin, D. M. , Clamp, M. , & Barton, G. J. (2009). Jalview Version 2—A multiple sequence alignment editor and analysis workbench. Bioinformatics, 25(9), 1189–1191. 10.1093/bioinformatics/btp033 19151095 PMC2672624

[tpg220502-bib-0079] Xi, D. , Yin, T. , Han, P. , Yang, X. , Zhang, M. , Du, C. , Zhang, H. , & Liu, X. (2023). Genome‐wide identification of sweet orange *WRKY* transcription factors and analysis of their expression in response to infection by *Penicillium digitatum* . Current Issues in Molecular Biology, 45(2), 1250–1271. 10.3390/cimb45020082 36826027 PMC9954951

[tpg220502-bib-0080] Xu, Y. , Zhang, H. , Zhong, Y. , Jiang, N. , Zhong, X. , Zhang, Q. , Chai, S. , Li, H. , & Zhang, Z. (2022). Comparative genomics analysis of *bHLH* genes in cucurbits identifies a novel gene regulating cucurbitacin biosynthesis. Horticulture Research, 9, uhac038. 10.1093/hr/uhac038 35184192 PMC9071377

[tpg220502-bib-0081] Yang, T. , Yao, S. , Hao, L. , Zhao, Y. , Lu, W. , & Xiao, K. (2016). Wheat bHLH‐type transcription factor gene *TabHLH1* is crucial in mediating osmotic stresses tolerance through modulating largely the ABA‐associated pathway. Plant Cell Reports, 35(11), 2309–2323. 10.1007/s00299-016-2036-5 27541276

[tpg220502-bib-0082] Yang, X. , Jia, Z. , Pu, Q. , Tian, Y. , Zhu, F. , & Liu, Y. (2022). ABA mediates plant development and abiotic stress via alternative splicing. International Journal of Molecular Sciences, 23(7), 3796. 10.3390/ijms23073796 35409156 PMC8998868

[tpg220502-bib-0083] Yanhui, C. , Xiaoyuan, Y. , Kun, H. , Meihua, L. , Jigang, L. , Zhaofeng, G. , Zhiqiang, L. , Yunfei, Z. , Xiaoxiao, W. , Xiaoming, Q. , Yunping, S. , Li, Z. , Xiaohui, D. , Jingchu, L. , Xing‐Wang, D. , Zhangliang, C. , Hongya, G. , & Li‐Jia, Q. (2006). The MYB transcription factor superfamily of *Arabidopsis*: Expression analysis and phylogenetic comparison with the rice MYB family. Plant Molecular Biology, 60(1), 107–124. 10.1007/s11103-005-2910-y 16463103

[tpg220502-bib-0084] Yin, T. , Han, P. , Xi, D. , Yu, W. , Zhu, L. , Du, C. , Yang, N. , Liu, X. , & Zhang, H. (2022). Genome‐wide identification, characterization, and expression profile of *NBS‐LRR*gene family in sweet orange (*Citrus sinensis*). Gene, 854, 147117. 10.1016/j.gene.2022.147117 36526123

[tpg220502-bib-0085] Yu, Z. , Duan, X. , Luo, L. , Dai, S. , Ding, Z. , & Xia, G. (2020). How plant hormones mediate salt stress responses. Trends in Plant Science, 25(11), 1117–1130. 10.1016/j.tplants.2020.06.008 32675014

[tpg220502-bib-0086] Zhang, A. , Yang, X. , Lu, J. , Song, F. , Sun, J. , Wang, C. , Lian, J. , Zhao, L. , & Zhao, B. (2021). OsIAA20, an Aux/IAA protein, mediates abiotic stress tolerance in rice through an ABA pathway. Plant Science, 308, 110903. 10.1016/j.plantsci.2021.110903 34034863

[tpg220502-bib-0087] Zhang, F. , Yao, J. , Ke, J. , Zhang, L. , Lam, V. Q. , Xin, X. F. , Zhou, X. E. , Chen, J. , Brunzelle, J. , Griffin, P. R. , Zhou, M. , Xu, H. E. , Melcher, K. , & He, S. Y. (2015). Structural basis of JAZ repression of MYC transcription factors in jasmonate signalling. Nature, 525(7568), 269–273. 10.1038/nature14661 26258305 PMC4567411

[tpg220502-bib-0088] Zhao, K. , Li, S. , Yao, W. , Zhou, B. , Li, R. , & Jiang, T. (2018). Characterization of the basic helix‐loop‐helix gene family and its tissue‐differential expression in response to salt stress in poplar. PeerJ, 6, e4502. 10.7717/peerj.4502 29576971 PMC5857177

[tpg220502-bib-0089] Zhao, S. , Zhang, Q. , Liu, M. , Zhou, H. , Ma, C. , & Wang, P. (2021). Regulation of plant responses to salt stress. International Journal of Molecular Sciences, 22(9), 4609. 10.3390/ijms22094609 33924753 PMC8125386

[tpg220502-bib-0090] Zhou, M. , & Memelink, J. (2016). Jasmonate‐responsive transcription factors regulating plant secondary metabolism. Biotechnology Advances, 34(4), 441–449. 10.1016/j.biotechadv.2016.02.004 26876016

[tpg220502-bib-0091] Zhou, X. , Liao, Y. , Kim, S. U. , Chen, Z. , Nie, G. , Cheng, S. , Ye, J. , & Xu, F. (2020). Genome‐wide identification and characterization of *bHLH* family genes from *Ginkgo biloba* . Scientific Reports, 10(1), Article 13723. 10.1038/s41598-020-69305-3 32792673 PMC7426926

[tpg220502-bib-0092] Zhu, K. , Chen, H. , Zhang, Y. , Liu, Y. , Zheng, X. , Xu, J. , Ye, J. , & Deng, X. (2022). Carotenoid extraction, detection, and analysis in citrus. Methods in Enzymology, 670, 179–212. 10.1016/bs.mie.2022.01.006 35871836

[tpg220502-bib-0093] Zhu, L. , Yin, T. , Zhang, M. , Yang, X. , Wu, J. , Cai, H. , Yang, N. , Li, X. , Wen, K. , Chen, D. , Zhang, H. , & Liu, X. (2024). Genome‐wide identification and expression pattern analysis of the kiwifruit GRAS transcription factor family in response to salt stress. BMC Genomics, 25, Article 12. 10.1186/s12864-023-09915-z 38166720 PMC10759511

