# Supplementary material for: Identification of the sweet orange (Citrus sinensis) bHLH gene family and the role of CsbHLH55 and CsbHLH87 in regulating salt stress
Source: Plant Genome. 2024 Aug 31;17(3):e20502. doi: 10.1002/tpg2.20502 (PMC12807166; doi:10.1002/tpg2.20502)
Supplement: Supplementary file 1 [file TPG2-17-e20502-s001.docx]

**Fig. S1.** Correlation coefficient plot of qRT-PCR results with FPKM values of transcriptome data. Different colors indicate the correlation coefficients of qRT-PCR results of different genes with FPKM values of transcriptome data.

**Fig. S2.** Heatmap of expression of six differentially expressed genes.

**Fig. S3.** Flowchart of ABA-dependent or non-dependent pathways encoding *CsbHLHs*.

**Table S1** Gene IDs and names of *Arabidopsis thaliana* bHLH genes.

| AtbHLH ID | AtbHLH name |
| --- | --- |
| AT5G41315 | AtbHLH1 |
| AT2G31220 | AtbHLH10 |
| AT2G41240 | AtbHLH100 |
| AT5G04150 | AtbHLH101 |
| AT1G69010 | AtbHLH102 |
| AT4G21340 | AtbHLH103 |
| AT4G14410 | AtbHLH104 |
| AT5G54680 | AtbHLH105 |
| AT2G41130 | AtbHLH106 |
| AT3G56770 | AtbHLH107 |
| AT1G25310 | AtbHLH108 |
| AT1G68240 | AtbHLH109 |
| AT4G36060 | AtbHLH11 |
| AT1G27660 | AtbHLH110 |
| AT1G31050 | AtbHLH111 |
| AT1G61660 | AtbHLH112 |
| AT3G19500 | AtbHLH113 |
| AT4G05170 | AtbHLH114 |
| AT1G51070 | AtbHLH115 |
| AT3G26744 | AtbHLH116 |
| AT3G22100 | AtbHLH117 |
| AT4G25400 | AtbHLH118 |
| AT4G28811 | AtbHLH119 |
| AT4G00480 | AtbHLH12 |
| AT5G51790 | AtbHLH120 |
| AT3G19860 | AtbHLH121 |
| AT1G51140 | AtbHLH122 |
| AT3G20640 | AtbHLH123 |
| AT2G46970 | AtbHLH124 |
| AT1G62975 | AtbHLH125 |
| AT4G25410 | AtbHLH126 |
| AT4G28815 | AtbHLH127 |
| AT1G05805 | AtbHLH128 |
| AT2G43140 | AtbHLH129 |
| AT1G01260 | AtbHLH13 |
| AT2G42280 | AtbHLH130 |
| AT3G62090 | AtbHLH132 |
| AT5G15160 | AtbHLH134 |
| AT1G74500 | AtbHLH135 |
| AT5G39860 | AtbHLH136 |
| AT5G50915 | AtbHLH137 |
| AT2G31215 | AtbHLH138 |
| AT5G43175 | AtbHLH139 |
| AT4G00870 | AtbHLH14 |
| AT5G38860 | AtbHLH141 |
| AT5G64340 | AtbHLH142 |
| AT5G09460 | AtbHLH143 |
| AT1G29950 | AtbHLH144 |
| AT5G50010 | AtbHLH145 |
| AT4G30180 | AtbHLH146 |
| AT3G17100 | AtbHLH147 |
| AT3G06590 | AtbHLH148 |
| AT1G09250 | AtbHLH149 |
| AT2G20180 | AtbHLH15 |
| AT3G05800 | AtbHLH150 |
| AT2G47270 | AtbHLH151 |
| AT1G22380 | AtbHLH152 |
| AT1G05710 | AtbHLH153 |
| AT2G31730 | AtbHLH154 |
| AT2G31280 | AtbHLH155 |
| AT2G27230 | AtbHLH156 |
| AT1G64625 | AtbHLH157 |
| AT2G43060 | AtbHLH158 |
| AT4G30410 | AtbHLH159 |
| AT4G00050 | AtbHLH16 |
| AT1G71200 | AtbHLH160 |
| AT3G47710 | AtbHLH161 |
| AT4G20970 | AtbHLH162 |
| AT2G46510 | AtbHLH17 |
| AT2G22750 | AtbHLH18 |
| AT2G22760 | AtbHLH19 |
| AT1G63650 | AtbHLH2 |
| AT2G22770 | AtbHLH20 |
| AT2G16910 | AtbHLH21 |
| AT4G21330 | AtbHLH22 |
| AT4G28790 | AtbHLH23 |
| AT4G36930 | AtbHLH24 |
| AT4G37850 | AtbHLH25 |
| AT1G02340 | AtbHLH26 |
| AT4G29930 | AtbHLH27 |
| AT5G46830 | AtbHLH28 |
| AT2G28160 | AtbHLH29 |
| AT4G16430 | AtbHLH3 |
| AT1G68810 | AtbHLH30 |
| AT1G59640 | AtbHLH31 |
| AT3G25710 | AtbHLH32 |
| AT1G12860 | AtbHLH33 |
| AT3G23210 | AtbHLH34 |
| AT5G57150 | AtbHLH35 |
| AT5G51780 | AtbHLH36 |
| AT3G50330 | AtbHLH37 |
| AT3G56970 | AtbHLH38 |
| AT3G56980 | AtbHLH39 |
| AT4G17880 | AtbHLH4 |
| AT4G00120 | AtbHLH40 |
| AT5G56960 | AtbHLH41 |
| AT4G09820 | AtbHLH42 |
| AT5G09750 | AtbHLH43 |
| AT1G18400 | AtbHLH44 |
| AT3G06120 | AtbHLH45 |
| AT5G08130 | AtbHLH46 |
| AT3G47640 | AtbHLH47 |
| AT2G42300 | AtbHLH48 |
| AT1G68920 | AtbHLH49 |
| AT5G46760 | AtbHLH5 |
| AT1G73830 | AtbHLH50 |
| AT2G40200 | AtbHLH51 |
| AT1G30670 | AtbHLH52 |
| AT2G34820 | AtbHLH53 |
| AT1G27740 | AtbHLH54 |
| AT1G12540 | AtbHLH55 |
| AT4G28800 | AtbHLH56 |
| AT4G01460 | AtbHLH57 |
| AT4G36540 | AtbHLH58 |
| AT4G02590 | AtbHLH59 |
| AT1G32640 | AtbHLH6 |
| AT3G57800 | AtbHLH60 |
| AT5G10570 | AtbHLH61 |
| AT3G07340 | AtbHLH62 |
| AT4G34530 | AtbHLH63 |
| AT2G18300 | AtbHLH64 |
| AT3G59060 | AtbHLH65 |
| AT2G24260 | AtbHLH66 |
| AT3G61950 | AtbHLH67 |
| AT4G29100 | AtbHLH68 |
| AT4G30980 | AtbHLH69 |
| AT1G03040 | AtbHLH7 |
| AT2G46810 | AtbHLH70 |
| AT5G46690 | AtbHLH71 |
| AT5G61270 | AtbHLH72 |
| AT5G67110 | AtbHLH73 |
| AT1G10120 | AtbHLH74 |
| AT1G25330 | AtbHLH75 |
| AT1G26260 | AtbHLH76 |
| AT3G23690 | AtbHLH77 |
| AT5G48560 | AtbHLH78 |
| AT5G62610 | AtbHLH79 |
| AT1G09530 | AtbHLH8 |
| AT1G35460 | AtbHLH80 |
| AT4G09180 | AtbHLH81 |
| AT5G58010 | AtbHLH82 |
| AT1G66470 | AtbHLH83 |
| AT2G14760 | AtbHLH84 |
| AT4G33880 | AtbHLH85 |
| AT5G37800 | AtbHLH86 |
| AT3G21330 | AtbHLH87 |
| AT5G67060 | AtbHLH88 |
| AT1G06170 | AtbHLH89 |
| AT2G43010 | AtbHLH9 |
| AT1G10610 | AtbHLH90 |
| AT2G31210 | AtbHLH91 |
| AT5G43650 | AtbHLH92 |
| AT5G65640 | AtbHLH93 |
| AT1G22490 | AtbHLH94 |
| AT1G72210 | AtbHLH96 |
| AT3G24140 | AtbHLH97 |
| AT5G53210 | AtbHLH98 |
| AT5G65320 | AtbHLH99 |

**Table S2** Design of qRT-PCR primers for eight CsbHLH genes in sweet orange.

| Gene name | Upstream primer (5'-3') | Downsream primer(5'-3') |
| --- | --- | --- |
| β-Actin | GTTCTTTCCCTTTATGCCAGT | TCCTTTGTTTATTCTCCACCTT |
| CsbHLH16 | GTGACGAGAAACAGAGGCTAA | GAGGAGGATGAGGTAAGAAGG |
| CsbHLH17 | CCGTAGCACCAAATGTAAGTAG | CTCCAGAAGAAATAATCCGAAC |
| CsbHLH49 | TGGGTTATTGGTGGTCATTAG | AGTAGGTTATCATTTGCATCCC |
| CsbHLH50 | TAGATGTTGGACCTTCACGAC | GATTGGCTTCTTTCTTGCTTT |
| CsbHLH55 | CTTTGGTGTGGCTAACTGG | CTTGACTTGGTGGACGAGA |
| CsbHLH87 | AGGAGAAGGAGAGAAAGGAAA | TCATCAACAGCATCATCAGTC |

**Table S3** Analysis of physicochemical properties of CsbHLH TFs.

| Gene name | Gene ID | Number_of_amine_acid | Molecular_weight(KDa) | Theoretical_pi | Formula | Total_number_of_atoms | Instability_index | Aliphatic_index |
| --- | --- | --- | --- | --- | --- | --- | --- | --- |
| CsbHLH1 | Cs_ont_1g004730 | 565 | 61.69 | 9.01 | C_2656_H_4232_N_786_O_873_S_17_ | 8564 | 52.29 | 56.81 |
| CsbHLH2 | Cs_ont_1g017040 | 340 | 37.95 | 5.45 | C_1602_H_2531_N_483_O_553_S_17_ | 5186 | 64.05 | 55.38 |
| CsbHLH3 | Cs_ont_1g020790. | 340 | 38.09 | 6.35 | C_1627_H_2630_N_486_O_540_S_14_ | 5297 | 58.09 | 67.44 |
| CsbHLH4 | Cs_ont_1g021700 | 292 | 31.48 | 5 | C_1354_H_2137_N_383_O_457_S_12_ | 4343 | 61.88 | 70.17 |
| CsbHLH5 | Cs_ont_1g022070 | 409 | 44.40 | 6.41 | C_1885_H_3036_N_572_O_630_S_19_ | 6142 | 56.02 | 75.26 |
| CsbHLH6 | Cs_ont_1g022670 | 464 | 50.76 | 8.51 | C_2138_H_3458_N_644_O_696_S_45_ | 6981 | 60.71 | 55.13 |
| CsbHLH7 | Cs_ont_1g022810 | 252 | 28.75 | 6.33 | C_1281_H_2020_N_344_O_387_S_10_ | 4042 | 58.15 | 85.52 |
| CsbHLH8 | Cs_ont_1g022820 | 735 | 78.88 | 5.92 | C_3332_H_5326_N_996_O_1129_S_46_ | 10829 | 54.48 | 59.56 |
| CsbHLH9 | Cs_ont_1g024800 | 419 | 46.07 | 8.12 | C_1930_H_3106_N_596_O_641_S_36_ | 6309 | 62.1 | 55.27 |
| CsbHLH10 | Cs_ont_1g024900 | 949 | 104.55 | 8.63 | C_4552_H_7173_N_1315_O_1412_S_51_ | 14503 | 46.58 | 68.24 |
| CsbHLH11 | Cs_ont_2g002500 | 315 | 34.34 | 8.03 | C_1464_H_2351_N_435_O_478_S_20_ | 4748 | 58.35 | 69.05 |
| CsbHLH12 | Cs_ont_2g003830 | 337 | 36.93 | 5.07 | C_1599_H_2594_N_448_O_532_S_10_ | 5183 | 61.99 | 84.18 |
| CsbHLH13 | Cs_ont_2g004980 | 365 | 40.77 | 6.46 | C_1759_H_2825_N_531_O_561_S_12_ | 5688 | 68.26 | 77.29 |
| CsbHLH14 | Cs_ont_2g006370 | 548 | 59.99 | 6.55 | C_2570_H_4056_N_766_O_812_S_41_ | 8245 | 59.43 | 55.09 |
| CsbHLH15 | Cs_ont_2g006410 | 694 | 76.83 | 4.99 | C_3343_H_5298_N_952_O_1087_S_19_ | 10699 | 64.34 | 77.81 |
| CsbHLH16 | Cs_ont_2g006550 | 519 | 58.22 | 6.33 | C_2551_H_4019_N_735_O_787_S_20_ | 8112 | 39.45 | 78.81 |
| CsbHLH17 | Cs_ont_2g008620 | 437 | 49.59 | 5.92 | C_2184_H_3498_N_604_O_669_S_21_ | 6976 | 67.63 | 84.76 |
| CsbHLH18 | Cs_ont_2g011370 | 279 | 30.99 | 8.85 | C_1333_H_2171_N_399_O_424_S_13_ | 4340 | 50.87 | 81.18 |
| CsbHLH19 | Cs_ont_2g011730 | 613 | 68.14 | 5.95 | C_2969_H_4709_N_867_O_934_S_20_ | 9499 | 39.94 | 77.62 |
| CsbHLH20 | Cs_ont_2g019820 | 685 | 74.66 | 5.45 | C_3224_H_5095_N_917_O_1078_S_22_ | 10336 | 53.09 | 65.93 |
| CsbHLH21 | Cs_ont_2g025340 | 250 | 28.75 | 8.34 | C_1291_H_2064_N_338_O_386_S_8_ | 4087 | 48.53 | 88.56 |
| CsbHLH22 | Cs_ont_2g026680 | 317 | 35.82 | 5.19 | C_1550_H_2455_N_447_O_507_S_11_ | 4970 | 59.28 | 80 |
| CsbHLH23 | Cs_ont_2g030310.1 | 264 | 28.80 | 7.65 | C_1259_H_2044_N_358_O_397_S_8_ | 4066 | 50.42 | 83.94 |
| CsbHLH24 | Cs_ont_2g030320 | 502 | 55.66 | 6.16 | C_2436_H_3838_N_688_O_766_S_20_ | 7748 | 47.56 | 73.17 |
| CsbHLH25 | Cs_ont_2g030330 | 266 | 29.74 | 6.43 | C_1294_H_2061_N_373_O_401_S_15_ | 4144 | 55.01 | 75.9 |
| CsbHLH26 | Cs_ont_2g030340 | 532 | 60.49 | 5.91 | C_2666_H_4148_N_764_O_821_S_14_ | 8413 | 53.17 | 76.97 |
| CsbHLH27 | Cs_ont_2g030360 | 355 | 39.88 | 5.56 | C_1749_H_2723_N_499_O_541_S_15_ | 5527 | 48.54 | 78.85 |
| CsbHLH28 | Cs_ont_2g030380 | 1346 | 150.35 | 8.94 | C_6448_H_10435_N_1943_O_2086_S_56_ | 20968 | 61.88 | 63.05 |
| CsbHLH29 | Cs_ont_3g003260 | 363 | 40.54 | 6.12 | C_1767_H_2782_N_490_O_579_S_12_ | 5630 | 53.54 | 75.43 |
| CsbHLH30 | Cs_ont_3g005210 | 424 | 48.10 | 5.4 | C_2083_H_3306_N_610_O_664_S_18_ | 6681 | 57.49 | 76.34 |
| CsbHLH31 | Cs_ont_3g005310 | 515 | 57.14 | 5.38 | C_2475_H_3950_N_718_O_794_S_21_ | 7958 | 61.44 | 78.19 |
| CsbHLH32 | Cs_ont_3g005320 | 1142 | 127.69 | 8.83 | C_5691_H_9019_N_1569_O_1699_S_34_ | 18012 | 49.48 | 82.28 |
| CsbHLH33 | Cs_ont_3g005760 | 593 | 66.63 | 4.91 | C_2869_H_4560_N_826_O_932_S_34_ | 9221 | 38.65 | 77.89 |
| CsbHLH34 | Cs_ont_3g011440 | 310 | 34.64 | 6.6 | C_1522_H_2380_N_432_O_464_S_15_ | 4813 | 64.9 | 72 |
| CsbHLH35 | Cs_ont_3g011680 | 204 | 22.96 | 6.6 | C_989_H_1626_N_286_O_314_S_13_ | 3228 | 46.02 | 87.89 |
| CsbHLH36 | Cs_ont_3g011890 | 164 | 18.32 | 6.22 | C_785_H_1309_N_233_O_255_S_7_ | 2589 | 58.36 | 98.11 |
| CsbHLH37 | Cs_ont_3g012490 | 269 | 30.02 | 5.84 | C_1303_H_2118_N_374_O_415_S_11_ | 4221 | 53.87 | 92.42 |
| CsbHLH38 | Cs_ont_3g012990 | 512 | 57.85 | 9.2 | C_2532_H_4076_N_736_O_760_S_27_ | 8131 | 38.92 | 76.62 |
| CsbHLH39 | Cs_ont_3g013400 | 332 | 36.26 | 7.12 | C_1582_H_2527_N_447_O_500_S_14_ | 5070 | 55.09 | 73.73 |
| CsbHLH40 | Cs_ont_3g014080 | 316 | 35.29 | 8.37 | C_1557_H_2491_N_433_O_475_S_13_ | 4969 | 38.39 | 83.92 |
| CsbHLH41 | Cs_ont_3g016680 | 152 | 16.91 | 7.94 | C_731_H_1203_N_213_O_230_S_8_ | 2385 | 51.18 | 95.59 |
| CsbHLH42 | Cs_ont_3g019490 | 164 | 18.38 | 8.82 | C_790_H_1323_N_237_O_247_S_9_ | 2606 | 52.27 | 98.05 |
| CsbHLH43 | Cs_ont_3g027350 | 329 | 36.44 | 5.58 | C_1587_H_2503_N_453_O_508_S_12_ | 5063 | 67.77 | 75.96 |
| CsbHLH44 | Cs_ont_4g008480 | 196 | 22.00 | 8.67 | C_983_H_1610_N_270_O_285_S_7_ | 3155 | 53 | 112.35 |
| CsbHLH45 | Cs_ont_4g010930 | 459 | 51.59 | 6.16 | C_2279_H_3606_N_638_O_692_S_18_ | 7233 | 44.41 | 84.97 |
| CsbHLH46 | Cs_ont_4g016360 | 235 | 25.83 | 5.85 | C_1119_H_1803_N_321_O_356_S_12_ | 3611 | 57.26 | 72.72 |
| CsbHLH47 | Cs_ont_4g016860 | 626 | 70.24 | 5.65 | C_3065_H_4886_N_856_O_978_S_27_ | 9812 | 45.66 | 84.38 |
| CsbHLH48 | Cs_ont_4g022650 | 519 | 56.32 | 4.96 | C_2426_H_3900_N_682_O_814_S_21_ | 7843 | 51.52 | 80.42 |
| CsbHLH49 | Cs_ont_4g025270 | 445 | 49.46 | 7.08 | C_2149_H_3466_N_628_O_684_S_13_ | 6940 | 64.22 | 84.47 |
| CsbHLH50 | Cs_ont_4g028260 | 369 | 41.15 | 8.41 | C_1802_H_2893_N_499_O_572_S_14_ | 5780 | 49.51 | 77.97 |
| CsbHLH51 | Cs_ont_5g000010 | 212 | 24.01 | 9.66 | C_1054_H_1768_N_306_O_312_S_9_ | 3449 | 50.14 | 102.45 |
| CsbHLH52 | Cs_ont_5g001230 | 353 | 39.51 | 5.1 | C_1718_H_2726_N_482_O_554_S_16_ | 5496 | 63.42 | 78.5 |
| CsbHLH53 | Cs_ont_5g003140 | 169 | 19.01 | 7.07 | C_844_H_1295_N_231_O_255_S_8_ | 2633 | 56.25 | 60.65 |
| CsbHLH54 | Cs_ont_5g004630 | 2142 | 237.23 | 5.89 | C_10534_H_16896_N_2816_O_3181_S_104_ | 33531 | 40.79 | 98.81 |
| CsbHLH55 | Cs_ont_5g005120 | 469 | 49.18 | 5.83 | C_2062_H_3304_N_636_O_709_S_26_ | 6737 | 46.75 | 62.26 |
| CsbHLH56 | Cs_ont_5g005430 | 487 | 53.70 | 5.37 | C_2329_H_3684_N_670_O_749_S_20_ | 7452 | 45.14 | 75.34 |
| CsbHLH57 | Cs_ont_5g006010 | 253 | 27.66 | 6.13 | C_1177_H_1898_N_358_O_392_S_10_ | 3835 | 59.03 | 69.37 |
| CsbHLH58 | Cs_ont_5g010100 | 341 | 37.66 | 6.93 | C_1606_H_2591_N_477_O_537_S_15_ | 5226 | 60.58 | 55.81 |
| CsbHLH59 | Cs_ont_5g011220 | 402 | 45.61 | 4.8 | C_2010_H_3138_N_536_O_621_S_27_ | 6332 | 49.65 | 81.32 |
| CsbHLH60 | Cs_ont_5g011580 | 559 | 61.21 | 5.27 | C_2623_H_4155_N_771_O_868_S_26_ | 8443 | 46.09 | 71.36 |
| CsbHLH61 | Cs_ont_5g011730 | 313 | 35.04 | 4.92 | C_1534_H_2432_N_424_O_488_S_13_ | 4891 | 35.71 | 80.06 |
| CsbHLH62 | Cs_ont_5g013110 | 366 | 40.52 | 5.2 | C_1750_H_2738_N_496_O_586_S_13_ | 5583 | 54 | 66.67 |
| CsbHLH63 | Cs_ont_5g016910 | 278 | 30.03 | 5.52 | C_1274_H_2039_N_383_O_433_S_12_ | 4141 | 54.21 | 61.83 |
| CsbHLH64 | Cs_ont_5g019800 | 246 | 28.29 | 6.32 | C_1221_H_1971_N_357_O_393_S_11_ | 3953 | 71.94 | 81.59 |
| CsbHLH65 | Cs_ont_5g022670 | 312 | 34.67 | 6.31 | C_1506_H_2371_N_441_O_475_S_13_ | 4806 | 45.08 | 71.51 |
| CsbHLH66 | Cs_ont_5g032350 | 364 | 39.97 | 5.08 | C_1692_H_2686_N_498_O_593_S_15_ | 5484 | 61.76 | 66.51 |
| CsbHLH67 | Cs_ont_5g036600 | 238 | 26.93 | 7.01 | C_1169_H_1911_N_337_O_370_S_10_ | 3797 | 61.37 | 91.39 |
| CsbHLH68 | Cs_ont_5g038030 | 188 | 21.04 | 6.09 | C_903_H_1466_N_268_O_296_S_7_ | 2940 | 43.41 | 75.27 |
| CsbHLH69 | Cs_ont_5g039400 | 291 | 32.21 | 6.33 | C_1389_H_2189_N_399_O_459_S_12_ | 4448 | 43.7 | 63.44 |
| CsbHLH70 | Cs_ont_5g040940 | 177 | 19.65 | 6.96 | C_841_H_1406_N_246_O_273_S_10_ | 2776 | 66.03 | 96.21 |
| CsbHLH71 | Cs_ont_5g046050 | 236 | 25.95 | 8.36 | C_1139_H_1835_N_319_O_354_S_9_ | 3656 | 57.32 | 74.45 |
| CsbHLH72 | Cs_ont_5g046760 | 577 | 62.24 | 8.88 | C_2666_H_4263_N_793_O_894_S_16_ | 8632 | 52.34 | 67.44 |
| CsbHLH73 | Cs_ont_5g047480 | 245 | 27.73 | 6.18 | C_1202_H_1948_N_344_O_389_S_9_ | 3892 | 39.8 | 88.65 |
| CsbHLH74 | Cs_ont_5g047540 | 324 | 35.96 | 4.7 | C_1557_H_2466_N_432_O_527_S_9_ | 4991 | 37.15 | 78.89 |
| CsbHLH75 | Cs_ont_5g048050 | 294 | 31.30 | 5.85 | C_1368_H_2188_N_396_O_425_S_10_ | 4387 | 55.82 | 79.35 |
| CsbHLH76 | Cs_ont_5g050070 | 214 | 24.46 | 5.52 | C_1058_H_1682_N_308_O_339_S_10_ | 3397 | 63.87 | 67.1 |
| CsbHLH77 | Cs_ont_5g050360 | 649 | 70.79 | 7.91 | C_3008_H_4758_N_902_O_999_S_39_ | 9706 | 47.39 | 53.44 |
| CsbHLH78 | Cs_ont_6g001330 | 235 | 25.83 | 7.63 | C_1132_H_1815_N_321_O_347_S_11_ | 3626 | 51 | 75.49 |
| CsbHLH79 | Cs_ont_6g002740 | 181 | 20.57 | 9.13 | C_890_H_1476_N_262_O_273_S_11_ | 2912 | 45.93 | 94.75 |
| CsbHLH80 | Cs_ont_6g004050 | 431 | 48.77 | 4.88 | C_2119_H_3331_N_583_O_699_S_19_ | 6751 | 54 | 81.02 |
| CsbHLH81 | Cs_ont_6g005000.1 | 440 | 49.11 | 6.43 | C_2114_H_3353_N_625_O_686_S_19_ | 6797 | 55.24 | 65.41 |
| CsbHLH82 | Cs_ont_6g005750 | 252 | 28.57 | 6.16 | C_1236_H_1944_N_368_O_391_S_11_ | 3950 | 65.68 | 73.21 |
| CsbHLH83 | Cs_ont_6g014300 | 272 | 30.02 | 8.94 | C_1295_H_2063_N_383_O_409_S_15_ | 4165 | 49.32 | 67.17 |
| CsbHLH84 | Cs_ont_6g026370 | 267 | 29.84 | 9.38 | C_1304_H_2081_N_377_O_385_S_20_ | 4167 | 59.19 | 69.44 |
| CsbHLH85 | Cs_ont_7g001740 | 430 | 47.56 | 6.02 | C_2046_H_3206_N_612_O_669_S_15_ | 6548 | 48.4 | 65.81 |
| CsbHLH86 | Cs_ont_7g004080 | 553 | 60.02 | 6.06 | C_2560_H_4059_N_763_O_860_S_23_ | 8265 | 50.93 | 61.28 |
| CsbHLH87 | Cs_ont_7g010850 | 265 | 29.80 | 8.8 | C_1302_H_2095_N_377_O_397_S_13_ | 4184 | 40.89 | 78.79 |
| CsbHLH88 | Cs_ont_7g013200 | 370 | 41.63 | 6.14 | C_1801_H_2810_N_522_O_588_S_14_ | 5735 | 42.93 | 59.62 |
| CsbHLH89 | Cs_ont_7g013290 | 426 | 46.43 | 5.44 | C_1999_H_3162_N_568_O_657_S_24_ | 6410 | 50.74 | 64.08 |
| CsbHLH90 | Cs_ont_7g014320 | 558 | 59.59 | 6.1 | C_2541_H_4024_N_758_O_848_S_26_ | 8197 | 53.12 | 58.58 |
| CsbHLH91 | Cs_ont_7g015870 | 481 | 52.71 | 6.22 | C_2238_H_3598_N_662_O_752_S_28_ | 7278 | 48.75 | 67.3 |
| CsbHLH92 | Cs_ont_7g019890 | 181 | 20.68 | 9.17 | C_900_H_1485_N_261_O_273_S_11_ | 2930 | 41.61 | 90.39 |
| CsbHLH93 | Cs_ont_7g020580 | 139 | 15.64 | 5.8 | C_680_H_1123_N_191_O_213_S_8_ | 2215 | 46.14 | 101.58 |
| CsbHLH94 | Cs_ont_7g020750 | 406 | 44.88 | 6.41 | C_1925_H_3037_N_597_O_626_S_10_ | 6195 | 49.24 | 68.77 |
| CsbHLH95 | Cs_ont_7g024480 | 93 | 10.45 | 7.94 | C_436_H_748_N_140_O_150_S_3_ | 1477 | 93.56 | 101.72 |
| CsbHLH96 | Cs_ont_7g025520 | 363 | 41.52 | 8.91 | C_1796_H_2825_N_531_O_548_S_28_ | 5728 | 48.23 | 63.91 |
| CsbHLH97 | Cs_ont_7g026220 | 91 | 10.36 | 9.09 | C_440_H_755_N_139_O_142_S_3_ | 1479 | 84.39 | 108.24 |
| CsbHLH98 | Cs_ont_7g027630 | 242 | 26.89 | 6.39 | C_1155_H_1828_N_340_O_367_S_17_ | 3707 | 47.98 | 66.9 |
| CsbHLH99 | Cs_ont_7g028400 | 264 | 30.10 | 6.85 | C_1302_H_2065_N_371_O_417_S_16_ | 4171 | 59.09 | 63.9 |
| CsbHLH100 | Cs_ont_8g006110 | 656 | 73.46 | 5.11 | C_3226_H_5067_N_871_O_1020_S_34_ | 10218 | 54.09 | 82.65 |
| CsbHLH101 | Cs_ont_8g006120 | 290 | 32.91 | 6.32 | C_1426_H_2288_N_408_O_459_S_13_ | 4594 | 50.5 | 82.62 |
| CsbHLH102 | Cs_ont_8g006160 | 271 | 30.31 | 8.99 | C_1313_H_2151_N_377_O_421_S_11_ | 4273 | 42.54 | 79.45 |
| CsbHLH103 | Cs_ont_8g009230 | 92 | 10.36 | 6.21 | C_440_H_744_N_132_O_147_S_4_ | 1467 | 74.68 | 103.8 |
| CsbHLH104 | Cs_ont_8g012600 | 459 | 49.92 | 6.81 | C_2167_H_3323_N_629_O_709_S_12_ | 6840 | 61.01 | 53.77 |
| CsbHLH105 | Cs_ont_8g018940 | 892 | 102.05 | 6.76 | C_4594_H_7119_N_1223_O_1328_S_41_ | 14305 | 50.3 | 85.12 |
| CsbHLH106 | Cs_ont_8g021630 | 487 | 52.83 | 6.01 | C_2296_H_3528_N_642_O_752_S_21_ | 7239 | 60.47 | 54.76 |
| CsbHLH107 | Cs_ont_8g024860 | 292 | 31.19 | 5.01 | C_1341_H_2162_N_382_O_432_S_20_ | 4337 | 51.59 | 78.25 |
| CsbHLH108 | Cs_ont_8g026160.1 | 239 | 27.00 | 9.06 | C_1172_H_1918_N_336_O_371_S_11_ | 3808 | 64.21 | 78.74 |
| CsbHLH109 | Cs_ont_8g027350 | 748 | 82.35 | 5.68 | C_3577_H_5667_N_1005_O_1147_S_39_ | 11435 | 42.15 | 80.99 |
| CsbHLH110 | Cs_ont_8g028020. | 225 | 25.68 | 10.31 | C_1083_H_1831_N_363_O_341_S_9_ | 3627 | 69.11 | 74.13 |
| CsbHLH111 | Cs_ont_9g004690 | 431 | 46.49 | 7.66 | C_2018_H_3169_N_575_O_654_S_17_ | 6433 | 66.23 | 68.63 |
| CsbHLH112 | Cs_ont_9g007920 | 143 | 15.95 | 9.19 | C_706_H_1144_N_196_O_209_S_7_ | 2262 | 52.88 | 86.01 |
| CsbHLH113 | Cs_ont_9g013230 | 619 | 68.88 | 6.24 | C_3073_H_4787_N_855_O_920_S_14_ | 9649 | 50.57 | 87.16 |
| CsbHLH114 | Cs_ont_9g015330 | 339 | 36.87 | 6.42 | C_1615_H_2487_N_463_O_508_S_11_ | 5084 | 54.15 | 65.34 |
| CsbHLH115 | Cs_ont_9g016480 | 363 | 39.48 | 7.13 | C_1704_H_2664_N_500_O_549_S_17_ | 5434 | 68.76 | 61.32 |
| CsbHLH116 | Cs_ont_9g021680 | 372 | 40.71 | 4.57 | C_1773_H_2743_N_479_O_583_S_19_ | 5597 | 49.87 | 68.71 |
| CsbHLH117 | Cs_ont_9g023430 | 241 | 26.87 | 5.51 | C_1173_H_1806_N_330_O_374_S_11_ | 3694 | 54.49 | 61.45 |
| CsbHLH118 | Cs_ont_9g024620 | 491 | 54.63 | 6.58 | C_2383_H_3666_N_676_O_771_S_16_ | 7512 | 40.78 | 63.38 |
| CsbHLH119 | Cs_ont_9g024630 | 953 | 103.75 | 6.29 | C_4503_H_7128_N_1300_O_1435_S_41_ | 14407 | 37.74 | 77.43 |
| CsbHLH120 | Cs_ont_9g027180 | 852 | 94.58 | 4.92 | C_4109_H_6476_N_1134_O_1322_S_52_ | 13093 | 43.92 | 78.83 |

**Table S4** Structural domain prediction of CsbHLH28, 26, 63 and 99 genes.

| Gene ID | Gene name | Structural domain |
| --- | --- | --- |
| Cs_ont_2g030380 | CsbHLH28 | bHLH_SF superfamily |
| Cs_ont_2g030340 | CsbHLH26 | bHLH_SF superfamily |
| Cs_ont_5g016910 | CsbHLH63 | bHLH_SF superfamily |
| Cs_ont_7g028400 | CsbHLH99 | bHLH_SF superfamily |

**Table S5** GO enrichment analysis of sweet orange bHLH gene.

| CO ID | Term | Pvalue | All | Up-gene | Down-gene |
| --- | --- | --- | --- | --- | --- |
| GO:0010817 | regulation of hormone levels | 0.001687857 | 3 | 0 | 0 |
| GO:0009739 | response to gibberellin | 0.09174166 | 2 | 0 | 0 |
| GO:0009725 | response to hormone | 3.78744E-05 | 14 | 1 | 2 |
| GO:0009753 | response to jasmonic acid | 0.009778348 | 2 | 1 | 1 |
| GO:0006970 | response to osmotic stress | 0.05092416 | 3 | 0 | 2 |
| GO:0009737 | response to abscisic acid | 0.005840963 | 2 | 0 | 2 |
| GO:0009651 | response to salt stress | 0.079397999 | 3 | 0 | 2 |
| GO:0006952 | defense response | 5.58835E-06 | 3 | 0 | 0 |
| GO:0031347 | regulation of defense response | 0.000276667 | 1 | 0 | 0 |
| GO:0006972 | hyperosmotic response | 0.597930801 | 2 | 0 | 1 |
| GO:0042538 | hyperosmotic salinity response | 0.436459411 | 2 | 0 | 1 |
| GO:0042445 | hormone metabolic process | 0.002995427 | 3 | 0 | 0 |
| GO:0009755 | hormone-mediated signaling pathway | 0.004418826 | 4 | 1 | 0 |
| GO:0042446 | hormone biosynthetic process | 0.002084642 | 3 | 0 | 0 |
| GO:0009867 | jasmonic acid mediated signaling pathway | 0.006532266 | 1 | 0 | 0 |
| GO:0071470 | cellular response to osmotic stress | 0.782114639 | 1 | 1 | 0 |
| GO:0071215 | cellular response to abscisic acid stimulus | 0.176744852 | 1 | 0 | 1 |
| GO:0071472 | cellular response to salt stress | 0.572049913 | 1 | 0 | 1 |
| GO:0071369 | cellular response to ethylene stimulus | 0.23860925 | 3 | 0 | 1 |
| GO:0016020 | membrane | 4.46368E-07 | 1 | 0 | 0 |
| GO:0030117 | membrane coat | 0.561835972 | 1 | 0 | 0 |
| GO:0043226 | organelle | 0.999984478 | 1 | 0 | 0 |
| GO:0031090 | organelle membrane | 0.804378526 | 54 | 1 | 8 |
| GO:0043425 | bHLH transcription factor binding | 0.086481566 | 1 | 0 | 0 |
| GO:0005515 | protein binding | 0.717057731 | 3 | 0 | 1 |
| GO:0019904 | protein domain specific binding | 0.981780923 | 1 | 0 | 1 |
| GO:0016301 | kinase activity | 0.000635745 | 1 | 0 | 0 |

**Note:** Up -gene indicates the number of differentially significant up-regulated genes in the CsbHLH gene in salt stress, and Down -gene indicates the number of differentially significant down-regulated genes.

**Table S6** Physiological indexes of sweet orange seedlings analyzed under salt stress.

| Class | Serial number | Fresh weight（g） | Dry weight （g) | Relative water content (%) |
| --- | --- | --- | --- | --- |
| CK1 | 1 | 0.446 | 0.083 | 81.4 |
|  | 2 | 0.459 | 0.081 | 82.4 |
|  | 3 | 0.436 | 0.079 | 81.9 |
|  | 4 | 0.457 | 0.089 | 80.5 |
|  | 5 | 0.418 | 0.073 | 82.5 |
| CK2 | 6 | 0.396 | 0.071 | 82.1 |
|  | 7 | 0.389 | 0.07 | 82 |
|  | 8 | 0.438 | 0.083 | 81.1 |
|  | 9 | 0.429 | 0.077 | 82.1 |
|  | 10 | 0.383 | 0.075 | 80.4 |
| CK3 | 11 | 0.471 | 0.087 | 81.5 |
|  | 12 | 0.433 | 0.085 | 80.4 |
|  | 13 | 0.398 | 0.078 | 80.4 |
|  | 14 | 0.467 | 0.084 | 82 |
|  | 15 | 0.476 | 0.091 | 80.9 |
| T1 | 1 | 0.264 | 0.07 | 73.5 |
|  | 2 | 0.271 | 0.073 | 73.1 |
|  | 3 | 0.303 | 0.081 | 73.3 |
|  | 4 | 0.283 | 0.076 | 73.1 |
|  | 5 | 0.293 | 0.079 | 73 |
| T2 | 6 | 0.301 | 0.083 | 72.4 |
|  | 7 | 0.287 | 0.078 | 72.8 |
|  | 8 | 0.304 | 0.084 | 72.4 |
|  | 9 | 0.307 | 0.086 | 72 |
|  | 10 | 0.289 | 0.075 | 74 |
| T3 | 11 | 0.285 | 0.072 | 74.7 |
|  | 12 | 0.294 | 0.077 | 73.8 |
|  | 13 | 0.297 | 0.079 | 73.4 |
|  | 14 | 0.302 | 0.081 | 73.2 |
|  | 15 | 0.295 | 0.078 | 73.6 |

**Table S7** FPKM values of bHLH differentially significant genes in sweet orange under salt stress.

| Gene name | CK1:fpkm | CK2:fpkm | CK3:fpkm | T1:fpkm | T2:fpkm | T3:fpkm | CK_vs_Y_log2fc | CK_vs_Y_pval |
| --- | --- | --- | --- | --- | --- | --- | --- | --- |
| CsbHLH2 | 2.468747307 | 4.892580243 | 3.992518829 | 1.948025414 | 1.15136959 | 1.819620256 | -1.40212917 | 0.002817693 |
| CsbHLH14 | 6.336708876 | 8.847934596 | 11.26902962 | 3.352413068 | 4.069806029 | 3.234465265 | -1.518271915 | 0.000591223 |
| CsbHLH16 | 0.358493258 | 0.366388243 | 0.205053411 | 1.163840374 | 0.56649055 | 1.375796864 | 1.541120894 | 0.017501251 |
| CsbHLH17 | 0.19133022 | 0.952649409 | 0.208454019 | 1.143087248 | 2.687464439 | 2.619667891 | 2.146033499 | 0.002184332 |
| CsbHLH32 | 4.070488188 | 5.526224815 | 3.899076093 | 4.615445499 | 0.743214054 | 2.122918083 | -1.060090974 | 0.016898416 |
| CsbHLH45 | 13.52188182 | 6.757381881 | 19.1003029 | 2.055969242 | 3.002182703 | 2.79705358 | -2.567992183 | 0.002856999 |
| CsbHLH46 | 3.980016986 | 1.596253807 | 6.512589021 | 1.803697985 | 0.29929651 | 0.72688097 | -2.374626746 | 0.028928876 |
| CsbHLH49 | 328.6114172 | 174.8852304 | 394.3485371 | 67.46630055 | 12.11708487 | 19.15105919 | -3.44504048 | 1.25358E-05 |
| CsbHLH50 | 99.95469912 | 78.18663368 | 103.2891074 | 41.02942924 | 60.68040483 | 44.04204884 | -1.160347051 | 0.00087848 |
| CsbHLH51 | 3.539428912 | 2.750977001 | 4.436824559 | 1.632198453 | 2.224488904 | 2.001805893 | -1.094030961 | 0.014071447 |
| CsbHLH54 | 96.95441037 | 79.7396311 | 137.5502977 | 37.35716062 | 24.82862563 | 24.74056517 | -2.086545512 | 0.000107338 |
| CsbHLH55 | 5.261127859 | 7.5491927 | 3.878113318 | 2.612782795 | 2.470833191 | 2.647386892 | -1.28567325 | 0.002182308 |
| CsbHLH71 | 232.9550493 | 111.2233355 | 184.7212516 | 73.83907024 | 89.95738181 | 49.98111624 | -1.530445615 | 0.004353757 |
| CsbHLH75 | 0.354670583 | 0.39623471 | 0.526233666 | 0.909023799 | 1.327381495 | 1.81334288 | 1.450447184 | 0.005184757 |
| CsbHLH80 | 7.979150032 | 6.647993782 | 6.555389518 | 22.17337456 | 8.7369665 | 28.65588013 | 1.267779296 | 0.034558678 |
| CsbHLH87 | 92.75316076 | 70.98458139 | 120.0932082 | 45.96075746 | 39.97975317 | 44.6481658 | -1.350009608 | 0.002155911 |
| CsbHLH99 | 109.8947614 | 81.35000037 | 156.4655968 | 59.5235735 | 55.09843331 | 54.10955867 | -1.276323949 | 0.013804065 |

**Table S8** Raw CT values of qRT-PCR.

| Gene name | CK 1 | CK2 | CK3 | T1 | T2 | T3 |
| --- | --- | --- | --- | --- | --- | --- |
| β-Actin | 26.25 | 26.07 | 26.22 | 26.14 | 26.93 | 26.44 |
| CsbHLH16 | 22.61 | 22.00 | 22.31 | 22.37 | 21.31 | 22.34 |
| CsbHLH17 | 25.53 | 24.09 | 26.67 | 23.80 | 24.23 | 24.89 |
| CsbHLH49 | 17.81 | 21.38 | 18.03 | 22.05 | 20.12 | 18.64 |
| CsbHLH50 | 17.68 | 19.43 | 17.76 | 18.44 | 18.62 | 17.61 |
| CsbHLH55 | 23.94 | 23.07 | 24.96 | 24.69 | 24.96 | 24.12 |
| CsbHLH87 | 19.74 | 19.73 | 19.59 | 20.65 | 20.62 | 20.71 |

**Table S9** KEGG enrichment analysis of CsbHLH gene under salt stress.

| Gene name | KEGG term | Pvalue |
| --- | --- | --- |
| CsbHLH87 | Plant hormone signal transduction | 0.000777721 |
| CsbHLH87 | MAPK signaling pathway - plant | 0.009128862 |
| CsbHLH55 | Plant hormone signal transduction | 0.000777721 |
| CsbHLH55 | MAPK signaling pathway - plant | 0.009128862 |
| CsbHLH77 | Plant hormone signal transduction | 0.000777721 |
| CsbHLH77 | MAPK signaling pathway - plant | 0.009128862 |
| CsbHLH57 | Circadian rhythm - plant | 0.863312648 |
| CsbHLH57 | Plant hormone signal transduction | 0.000777721 |
| CsbHLH63 | Endocytosis | 0.182920178 |
| CsbHLH63 | Circadian rhythm - plant | 0.863312648 |
| CsbHLH63 | Plant hormone signal transduction | 0.000777721 |
| CsbHLH120 | MAPK signaling pathway - plant | 0.009128862 |
| CsbHLH84 | Circadian rhythm - plant | 0.863312648 |
| CsbHLH84 | Plant hormone signal transduction | 0.000777721 |
| CsbHLH90 | Plant hormone signal transduction | 0.000777721 |
| CsbHLH19 | Circadian rhythm - plant | 0.863312648 |
| CsbHLH19 | Plant hormone signal transduction | 0.000777721 |

**Table S10** Changes in phytohormone content under salt stress (14d).

| Accession | CK_Mean | CK_Median | CK_RSD | T_Mean | T_Median | T_RSD |
| --- | --- | --- | --- | --- | --- | --- |
| ABA | 3.83 | 4.54 | 37.59 | 48.84 | 48.78 | 10.32 |
| ACC | 25426.93 | 28846.21 | 49.53 | 7972.87 | 7787.61 | 11.16 |
| DHJA | 0.08 | 0.08 | 11.22 | 0.09 | 0.09 | 33.75 |
| GA19 | 1.44 | 1.31 | 15.31 | 0.03 | 0.03 | 0 |
| GA4 | 4.44 | 3.88 | 45.69 | 2.65 | 3.16 | 90.91 |
| GA7 | 21.09 | 23.98 | 32.2 | 21.16 | 20.51 | 8.77 |
| IAA | 2.55 | 2.61 | 6.31 | 4.37 | 4.16 | 25.97 |
| IBA | 11.79 | 14.52 | 81.09 | 8.58 | 9.16 | 38.11 |
| ICA | 14.76 | 15.96 | 16.57 | 28.61 | 19.94 | 70.5 |
| ICAId | 10.54 | 10.61 | 24.47 | 11.58 | 10.05 | 23.57 |
| IPA | 15.19 | 14.42 | 18.33 | 27.84 | 28.74 | 9.56 |
| JA | 216.19 | 248.12 | 36.57 | 7.24 | 5.91 | 78.53 |
| JA-Ile | 96.6 | 101.6 | 23.75 | 5.67 | 5.81 | 42.55 |
| Kt | 0.58 | 0.59 | 3.65 | 0.34 | 0.25 | 50.64 |
| MT | 0.51 | 0.51 | 3.58 | 0.03 | 0.03 | 0 |
| OPDA | 3019.16 | 2842.71 | 21.4 | 165.94 | 102.1 | 67.03 |
| SA | 14.71 | 9.55 | 87.01 | 10.9 | 7.58 | 68.52 |
| SAG | 327.99 | 289.58 | 29.93 | 423.7 | 436.44 | 41.21 |
| TZ | 0.54 | 0.61 | 32.96 | 0.5 | 0.52 | 22.72 |
| TZR | 2.09 | 2.04 | 26.98 | 4.49 | 4.32 | 26.72 |
